# Superimposed gratings induce diverse response patterns of gamma oscillations in primary visual cortex

**DOI:** 10.1038/s41598-021-83923-5

**Published:** 2021-03-02

**Authors:** Bin Wang, Chuanliang Han, Tian Wang, Weifeng Dai, Yang Li, Yi Yang, Guanzhong Yang, Lvyan Zhong, Yange Zhang, Yujie Wu, Gang Wang, Hongbo Yu, Dajun Xing

**Affiliations:** 1 State Key Laboratory of Cognitive Neuroscience and Learning and IDG/McGovern Institute for Brain Research, Beijing Normal University, Beijing, 100875 China; 2 Center of Brain Sciences, Beijing Institute of Basic Medical Sciences, Beijing, 100085 China; 3 Vision Research Laboratory, Center for Brain Science Research and School of Life Sciences, Fudan University, 2005 Songhu Road, Shanghai, 200433 China

**Keywords:** Neuroscience, Auditory system, Cellular neuroscience, Cognitive neuroscience, Visual system

## Abstract

Stimulus-dependence of gamma oscillations (GAMMA, 30–90 Hz) has not been fully understood, but it is important for revealing neural mechanisms and functions of GAMMA. Here, we recorded spiking activity (MUA) and the local field potential (LFP), driven by a variety of plaids (generated by two superimposed gratings orthogonal to each other and with different contrast combinations), in the primary visual cortex of anesthetized cats. We found two distinct narrow-band GAMMAs in the LFPs and a variety of response patterns to plaids. Similar to MUA, most response patterns showed that the second grating suppressed GAMMAs driven by the first one. However, there is only a weak site-by-site correlation between cross-orientation interactions in GAMMAs and those in MUAs. We developed a normalization model that could unify the response patterns of both GAMMAs and MUAs. Interestingly, compared with MUAs, the GAMMAs demonstrated a wider range of model parameters and more diverse response patterns to plaids. Further analysis revealed that normalization parameters for high GAMMA, but not those for low GAMMA, were significantly correlated with the discrepancy of spatial frequency between stimulus and sites’ preferences. Consistent with these findings, normalization parameters and diversity of high GAMMA exhibited a clear transition trend and region difference between area 17 to 18. Our results show that GAMMAs are also regulated in the form of normalization, but that the neural mechanisms for these normalizations might differ from those of spiking activity. Normalizations in different brain signals could be due to interactions of excitation and inhibitions at multiple stages in the visual system.

## Introduction

Gamma oscillation (30–90 Hz) is an outstanding feature of local field potentials (LFP) in the visual cortex^1–3^. It has been considered an important indicator of neural processing related to visual perception and cognition^4–8^, such as feature binding^9,10^ and information transfer between remote brain areas^11–14^. Although many studies have focused on the cognitive functions of gamma oscillations in the visual cortex, the characteristics of gamma oscillations remain less well-understood.

An important feature of gamma oscillations is that they can be effectively induced by drift gratings or moving bars^15,16^ and that their response properties change according to the grating parameters. For example, an increase in grating contrast leads to an increase in the amplitude and peak frequency of gamma oscillations^17,18^; a large-size drift grating induces stronger gamma oscillations than a smaller one^19,20^. Furthermore, additional contexts can also affect gamma oscillations. Studies have found that adding another grating orthogonal to the first grating results in decreased gamma oscillations^21,22^. To understand the above-described stimulus-dependence of gamma oscillations, one needs to know the response patterns of gamma oscillations as functions of these stimulus parameters. However, unlike the dependence of gamma oscillations on stimulus contrast and size, which have been well characterized^2,23^, how and why gamma oscillations change in association with changes in plaids (stimulus contexts) has not been systematically studied^21,22,24^.

The phenomenon of gamma oscillation suppression by the second superimposed grating is similar to that of cross-orientation suppression^8,25,26^ in spiking activity, which can be explained by the normalization model at both the individual neuron level^13,27–30^ and the population level^31^. Therefore, it is intriguing to know whether gamma oscillations and spiking activity have similar response patterns driven by plaids and whether these response patterns can be explained by a common form of normalization model.

In this paper, we recorded LFPs and multi-unit spiking activity (MUA) from the primary visual cortex (A17/A18) of anesthetized cats in response to various plaids formed by summing two orthogonal drift gratings with varying contrast. Subsequently, we compared the response patterns of gamma oscillations with those of MUA and quantified the cross-orientation interactions in the response patterns for gamma oscillations and MUA. We propose a descriptive model (modified normalization model) to capture the response patterns and their cross-orientation interactions for both gamma oscillations and MUA. Then we compared normalization parameters for low gamma, high gamma, and MUA in the model. The results show that the fitting parameters for gamma oscillations are different from and more diverse than those for MUA. The correlation between sites’ response properties (signal–noise ratio and two other factors related to specific spatial frequency and orientation) and the fitting parameters were studied. Finally, the region difference (between A17 and A18) for fitting parameters in the three signals was investigated.

## Materials and methods

### Preparation

All surgical and experimental procedures were carried out in accordance with the guidelines of The National Institutes of Health (USA), were carried out in compliance with the ARRIVE guidelines and were approved by the Institutional Animal Care and Use Committee of Beijing Normal University and Fudan University. Five adult cats (weighing 2–3.5 kg each) were injected with dexamethasone (0.4 mg/kg, subcutaneously) 12 h before the experiments. These cats were initially anesthetized with isoflurane gas (5% concentration) and were injected with atropine sulfate (0.05 mg/kg) and an antibiotic just before the anesthesia. The cats were then artificially ventilated through a tracheotomy. During the recordings and throughout the surgery, anesthesia was maintained with propofol (2–6 mg/kg/h), and paralysis was maintained with vecuronium bromide (0.1 mg/kg/h). The skull was secured to a metal rod for immobilization of the animals’ heads. The end-tidal CO_2_ was maintained at 3.5–4%, and body temperature was maintained at 37–38 °C using a feedback-controlled heating pad. Ventilation pressure, heart rate, electrocardiogram, and blood oxygen were monitored continuously throughout the experiment, as was the EEG recording. Eyes were treated with 1% atropine sulfate solution to dilate the pupils before being fitted with appropriate contact lenses and focused onto a tangent screen. During the whole visual experiment, the cats also wore an artificial pupil of 3-mm in diameter to restrict the amount of luminance. To maintain the cats’ health, they were injected with dexamethasone antibiotic every day (0.4 mg/kg) and with atropine sulfate (0.05 mg/kg) every other day.

### Recordings

Two settings of multi-electrode arrays were used to obtain simultaneous recordings from Area 17 and Area 18. One was a 10 × 10 grid of microelectrodes (1-mm electrode length, 0.4-mm electrode separation; Blackrock Microsystems), and the other was a 6 × 8 multi-electrode array (1-mmelectrode length, 0.4-mm inter-electrode spacing, and typical electrode impedances of a few hundred kiloohms at 1 kHz; Blackrock Microsystems). The array and the exposed cortex were covered in 1.5–2% agar to prevent the pulsation. Raw data was acquired using a Cerebus 128-channel system (Blackrock Microsystems). All LFP signals were low-pass filtered at 1000 Hz from the raw data and then down-sampled to 500 Hz. The MUAs were created by applying a voltage threshold^32^ (signal-to-noise ratio, 5.5) to the raw data. Both LFPs and MUAs were post-processed by removing channels that may not have been functional because of broken electrodes or noise.

### Visual stimulation

Visual stimuli were generated with a PC containing a Leadtek GeForce 6800 video cardand were presented monocularly on a CRT monitor (Dell P1230, refresh rate 100 Hz, mean luminance 32 cd/m^2^) placed 40 cm away from the cats’ eyes. We first mapped the receptive fields (RFs) of the whole recording sites for each experiment through a sparse noise experiment. Afterward, all stimuli were presented full-field to cover the RFs of all recording sites and were viewed monocularly. Typically, the size of the stimulus was chosen so that it formed the largest circle (38°) possible using the whole screen. The contrast of the gratings was modulated sinusoidally. Spatial frequency was adjusted to optimally drive gamma oscillations in LFP for most sites in the electrode array. Spatial frequencies of 0.03, 0.05, 0.07, 0.1, and 0.2 cycles/° were chosen for different cats (referred to as LM7, LM2, LM9, FM5, and EM7 respectively), to induce strong gamma oscillation. The time durations for the visual experiment were: pre-stimulus 0.4 s, on-stimulus 2 or 4 s, and off-stimulus 0.4 s. We used a stimulus setup that consisted of various plaids with varying contrasts. Grating contrasts of 0%, 7%, 10%, 14%, 21%, 31%, and 45% were used, and plaids were obtained by summing two gratings. For each experiment, the angle between component orientations in the plaid was fixed to 90°. All the stimuli were presented with a temporal frequency of 2 Hz. The stimuli were shown in random order in blocks presented at least ten times.

The spatial frequency for visual stimulus in plaid experiments was determined based on drifting grating experiments with different spatial frequencies. We on-line down-sampled and analyzed the LFP from 10 sites (out of 96 sites in total) from the array recording to get a rough estimation of the average preferred spatial frequency of MUA and that of gamma oscillation (between 60 and 100 Hz). The spatial frequency is chosen between these two mean values to activate most sites with strong gamma oscillation and MUA.

### Data analysis

#### Spectrum fitting

The power spectrum of the LFP response over 300–2000 ms (the same as in the previous work^25^) after the stimulus onset was estimated with multi-taper techniques^33^. This method was implemented on a Chronux toolbox (http://chronux.org/). A typical parameter setup (time-bandwidth product, 3; tapers, 5) was used to calculate the LFP spectrum. To quantify the responses, we separated the LFP spectrum into baseline power and narrow-band gamma oscillations. We used the following equations to capture the characteristics of these oscillations:

1 $$P\left(f\right)={w}_{b}F\left(f\right)+{w}_{{g}_{1}}G\left(f{\left|{u}_{1},\sigma \right.}_{1}\right)+{w}_{{g}_{2}}G\left(f{\left|{u}_{2},\sigma \right.}_{2}\right)+{k}_{0},$$

where,

2 $$F(f)=\frac{1}{(f-{f}_{0}{)}^{{n}_{0}}+{{b}_{0}}^{{n}_{0}}},$$

3 $$G(f{\left|{u}_{k},\sigma \right.}_{k})={e}^{-(f-{u}_{k}{)}^{2}/2{{\sigma }_{k}}^{2}},$$

With 30 Hz < ${u}_{1}$ < 100 Hz and 30 Hz < ${u}_{2}$ < 100 Hz. The variable $f$ represents the frequency bin in the spectrum. $F(f)$ is fitted to the broad-band oscillation that obeys a power law, and $G(f)$ is used for the narrow-band gamma oscillation that is the Gaussian function. ${w}_{b}$ and ${w}_{g}$ denote the weight of the baseline power and narrow-band oscillations respectively. ${\text{u}}_{\text{k}}$ ${u}_{k}$ and ${\sigma }_{k}$ represent the peak frequency and bandwidth of the gamma oscillation. ${f}_{0}$ is the initial peak frequency, and other parameters (${n}_{0}$*,* ${b}_{0}$, and ${k}_{0}$) are constant values. Note that in this paper, we used two Gaussian functions to capture the two gamma oscillations: we constrained fitting parameters ${u}_{k}$ (peak frequency) with restricted ranges for two gamma oscillations in each animal. Thus, the frequency range for low gamma would cover all sites’ lower peak frequencies under all conditions and the frequency range for high gamma will cover all sites’ higher peak frequencies under all conditions. Furthermore, the frequency ranges for high gamma and low gamma have no overlapping for each animal.

#### Criteria for choosing good gamma and MUA

The good MUA responses from the recording sites were selected according to their signal-to-noise ratio (SNR). The SNR was defined as the standard deviation of the stimulus MUA (the epoch from 200 ms after the stimulus onset to the end of the stimulus) divided by the standard deviation of the pre-stimulus MUA (epoch between the recording onset and stimulus onset). An MUA site was considered a good choice if the SNR of at least one stimulus condition at the site was greater than 2.5.

Strong narrow-band gamma oscillation sites were chosen through three steps. (1) Significant gamma oscillation sites were first identified. The z-score of the power spectrum was computed relative to the spontaneous activity. For each frequency bin and stimulus condition, the power spectrum of the baseline activity (the trials during the blank screen) was subtracted from that of the induced activity and divided by the standard deviation of the baseline activity (considering trials of all stimulus conditions). A recording site was considered to have significant gamma oscillation if at least one bin in the frequency range between 30 and 98 Hz showed a z-score value greater than 1.96 (95% threshold) for the situation where the drifting grating evoked the highest firing rate. (2) For the significant gamma sites, we then fitted the spectrum using Eqs. (1)–(3). We evaluated the fitting spectrum using the goodness of fit, and only the sites where the goodness of fit was greater than 0.8 were selected as good fitting sites. (3) For these good fitting sites, the relative fitting gamma power (Gamma SNR) was calculated as the narrow-band oscillation weight ${w}_{g}$ under stimulus condition divided by ${w}_{g}$ under blank condition. A site was considered a good choice of gamma oscillation if the Gamma SNR of at least one stimulus condition at the site was greater than 2.

However, the response matrix consisted of 49 visual responses induced by the corresponding stimuli. The site selections introduced above could not guarantee that the response matrix had a good pattern. Therefore, before the pattern analysis, we introduced a method to determine whether a site had a good response pattern. We assumed that if a site had a good response pattern, its responses to drifting gratings at different contrasts must be well explained by a contrast response function described by Eq. (4); and if a site’s contrast response tuning couldn’t be well fitted by Eq. (4), we excluded the signal from the site for further analysis. The contrast response was fitted by the following model:

4 $$\text{R}\left(\text{c}\right)={R}_{max}\cdot \frac{{c}^{{n}_{1}}}{{{c}_{50}}^{{n}_{2}}+{c}^{{n}_{2}}}+{R}_{0},$$

where c denotes the contrast value of the grating, c_50_ is half-maximal contrast; n_1_ and ${\text{R}}_{2}$ n_2_ are constant exponents related to response normalization. R_*max*_ and R_0_ are the estimations of the maximum and initial responses respectively.

The goodness of fit for the result was defined as Eq. (5). Only the site had goodness of fit larger than 0.6 was chosen for further analysis.

5 $$\text{Goodness of fit}=1-\frac{\sum_{1}^{n}{{(R}_{data}\left(i\right)-{R}_{model}\left(i\right))}^{2}}{\sum_{1}^{n}{({R}_{data}\left(i\right)-\sum_{1}^{n}{R}_{data}\left(i\right)/n)}^{2}},$$

In summary, sites with good MUA were selected by satisfying both the MUA SNR criteria and the good response pattern criteria; sites with good gamma were selected by satisfying both the strong gamma oscillation criteria and the good response pattern criteria; the sites with all good signals refer to those sites with both good MUA and good gamma (the chosen data set was summarized in Table 1).

**Table 1:** Fundamental information for the five cats.

	EM7	FM5	LM2	LM7	LM9
Size	38^o^	38^o^	38^o^	38^o^	38^o^
SF (cycle/°)	0.2	0.1	0.05	0.03	0.07
Orientations	0°, 90^o^	30 ^o^, 120^o^	30°, 120^o^	30°, 120^o^	0°, 90^o^
Sites with good MUA	65 (67.8%)	37 (38.5%)	23 (24%)	19 (19.8%)	39 (40.6%)
Sites with good gamma	79 (82.3%)	91 (94.8%)	90 (93.8%)	44 (45.8%)	92 (95.8%)
Sites with all signals good	52 (54.2%)	24 (25%)	18 (18.8%)	14 (14.6%)	30 (31.2%)

The first 3 rows represent the main stimulus parameters (size, spatial frequency, and orientation) used in plaid experiments. Row 1: We used large size (38° represents the largest circle on our screen) stimulus to activate good gamma and MUA response; Row 2: The spatial frequency used in the plaid experiment for 5 animals; Row 3: Two orthogonal orientations used in the plaid experiment are listed for each animal. The other 3 rows show the numbers of sites with good MUA, good gamma, and good signals for both respectively.

#### Response pattern analysis

To depict the response pattern, we proposed an index to capture the characteristics of the patterns of gamma oscillation and MUA. The interaction index is defined as Eqs. (6) and (7), which can describe the level of cross-orientation interaction.

6 $$\text{Interaction Index}= 2\cdot \frac{\sum_{{c}_{1}>0,{c}_{2}>0}{M}_{1}\left({c}_{1},{c}_{2}\right)-\sum_{{c}_{1}>0,{c}_{2}>0}{M}_{2}({c}_{1},{c}_{2})}{\sum_{{c}_{1}>0,{c}_{2}>0}{M}_{1}\left({c}_{1},{c}_{2}\right)+\sum_{{c}_{1}>0,{c}_{2}>0}{M}_{2}({c}_{1},{c}_{2})},$$

7 $${M}_{2}\left({\text{c}}_{1},{\text{c}}_{2}\right)={M}_{1}\left({\text{c}}_{1},0\right)+{M}_{1}\left(0,{\text{c}}_{2}\right)-{M}_{1}({0,0}),$$

The *c*_1_ and *c*_2_ denote the contrast values of the two orthogonal gratings. Considering the generation of a plaid that is the summation of two-component gratings orthogonal to each other, a prediction matrix (*M*_2_) is proposed. The interaction index is then defined as the difference between the prediction matrix and the raw response matrix (*M*_1_). If the index is negative, the plaid part of the raw input matrix is smaller than the linear prediction matrix, and the changing pattern of the raw response matrix tends to behave like the cross-orientation suppression phenomenon.

#### Statistical analysis

A non-parametric test (Bootstrap method^34^) was used to do the statistical analysis for the results in this paper. The bootstrap method was implemented by the following steps: (1) we randomly selected N samples with replacement from the raw data set (N is the number of trial in the dataset); (2) we then calculated the value we want to test (mean value or correlation coefficient for example) for the selected data; (3) we repeated step (1) and (2) for 1000 times and counted the number (K) of these mean values that meet the test. We then calculated 1-K/B as the p-value. Only if 1-K/B is lower than 0.05, we considered that the mean value of the raw dataset is significantly different from the expectation of the test. The equivalent p-value for the bootstrap method is calculated as 1-K/B. The statistical differences between the mean value of the interaction index (for gamma oscillations and MUA) and zero were calculated through this method. Similarly, the bootstrap method was also applied to the delta interaction index (fit response minus real response). We used the bootstrap method to compare the fitting parameters of low gamma and high gamma (or other pairs of signals: low gamma and MUA; high gamma and MUA) in the normalization model. Pearson’s correlation was used to quantify the linear correlation between the interaction index of one signal (low gamma, high gamma, and MUA) and another signal (low gamma, high gamma, and MUA) different from the first one. Robust regression analysis for the correlation measurements was also examined. To evaluate the performance of the modified normalization model for a given signal, the linear correlation between the fit and real response was calculated through Pearson’s correlation. Note that if Pearson’s correlation is not suitable, Spearman’s rank correlation coefficient was used instead.

## Results

**Figure 1:**
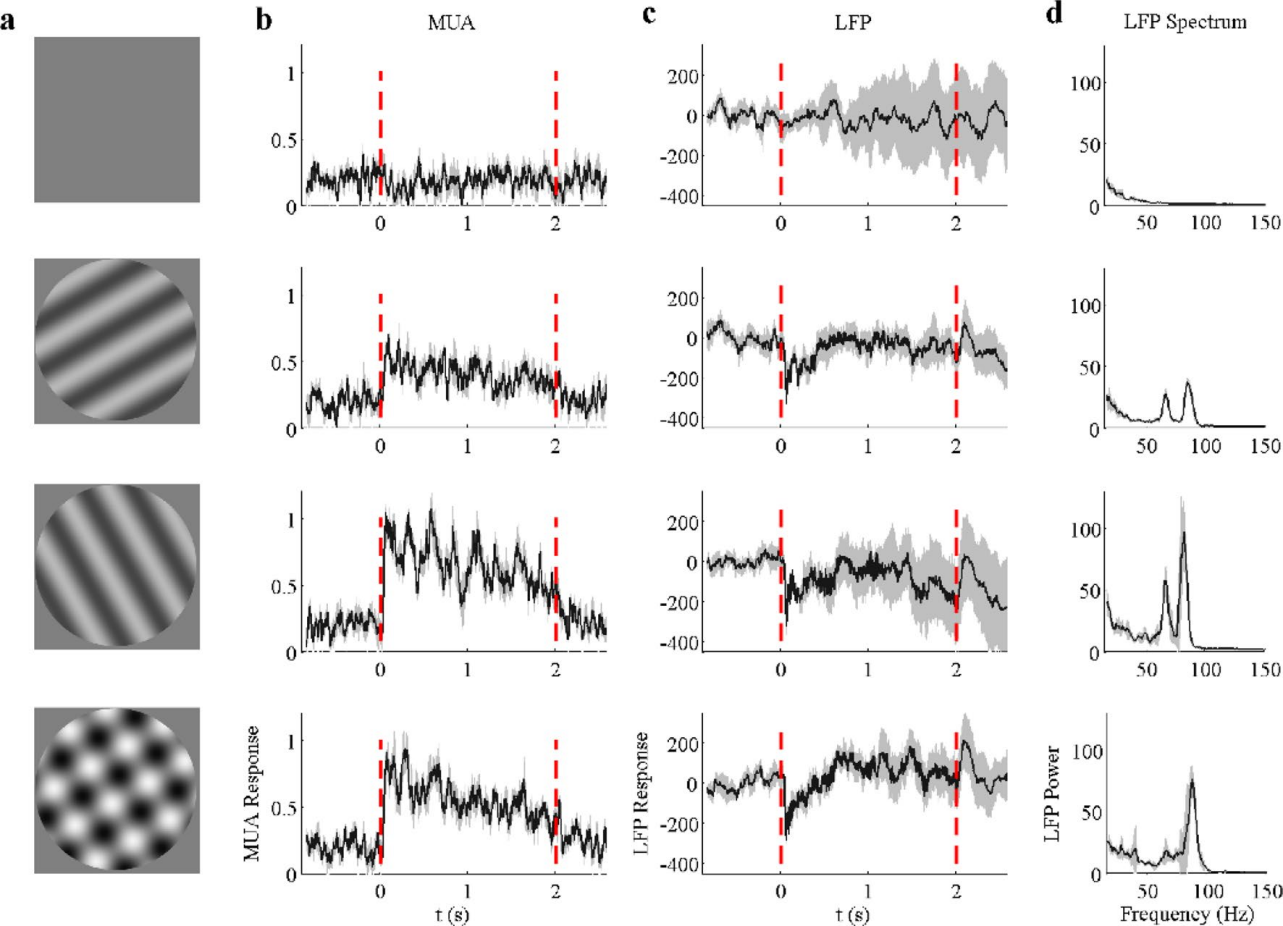
Neural responses are driven by a blank, two single gratings, and a plaid (superimposed gratings) for an example site. (**a**) demonstrates the visual stimuli (from top to bottom): grey screen, drift grating, another drift grating orthogonal to the previous one, and a plaid formed by the summation of the above two drift gratings. Note that the stimuli were presented during the time period marked by the two red dot lines in (**b**) and (**c**). (**b**) shows trial averaged (n = 10) spiking activity (MUA) driven by the four different stimuli shown in (**a**). (**c**) shows trial averaged (n = 10) LFP responses driven by the stimuli shown in (**a**). (**d**) presents the visually induced LFP power spectrum (calculated through the time course from 0.3 to 2 s, 10 trials averaged). The mean value is labeled as black solid line and 95% confidence interval is labeled as gray zone.

MUA and LFPs were acquired from 96-electrode arrays or two 48-electrode arrays implanted in five anesthetized cats (named EM7, FM5, LM2, LM7, and LM9). The arrays covered an area of 16 mm^2^, which contained brain areas 17 and 18. Large-sized (38°) visual stimuli (blank, two drift gratings, and plaid) were presented (drifting at 2 Hz per cycle and lasting for 2 s) on the screen to cover the receptive fields of all the recording sites of these cats (Fig. 1a). The stimulus orientation and spatial frequency of the drifting gratings were chosen to be optimal for most sites in each array. In total, 480 sites were recorded. We obtained 183 sites with good MUA and 396sites that had strong gamma oscillations in the LFPs; And 138 sites have both good MUA and good gamma based on selection criteria (detailed in “Materials and methods”). The chosen stimulus parameters for the five cats are listed in Table 1.

### Two distinct narrow-band gamma oscillations coexist in the cat visual cortex

**Figure 2:**
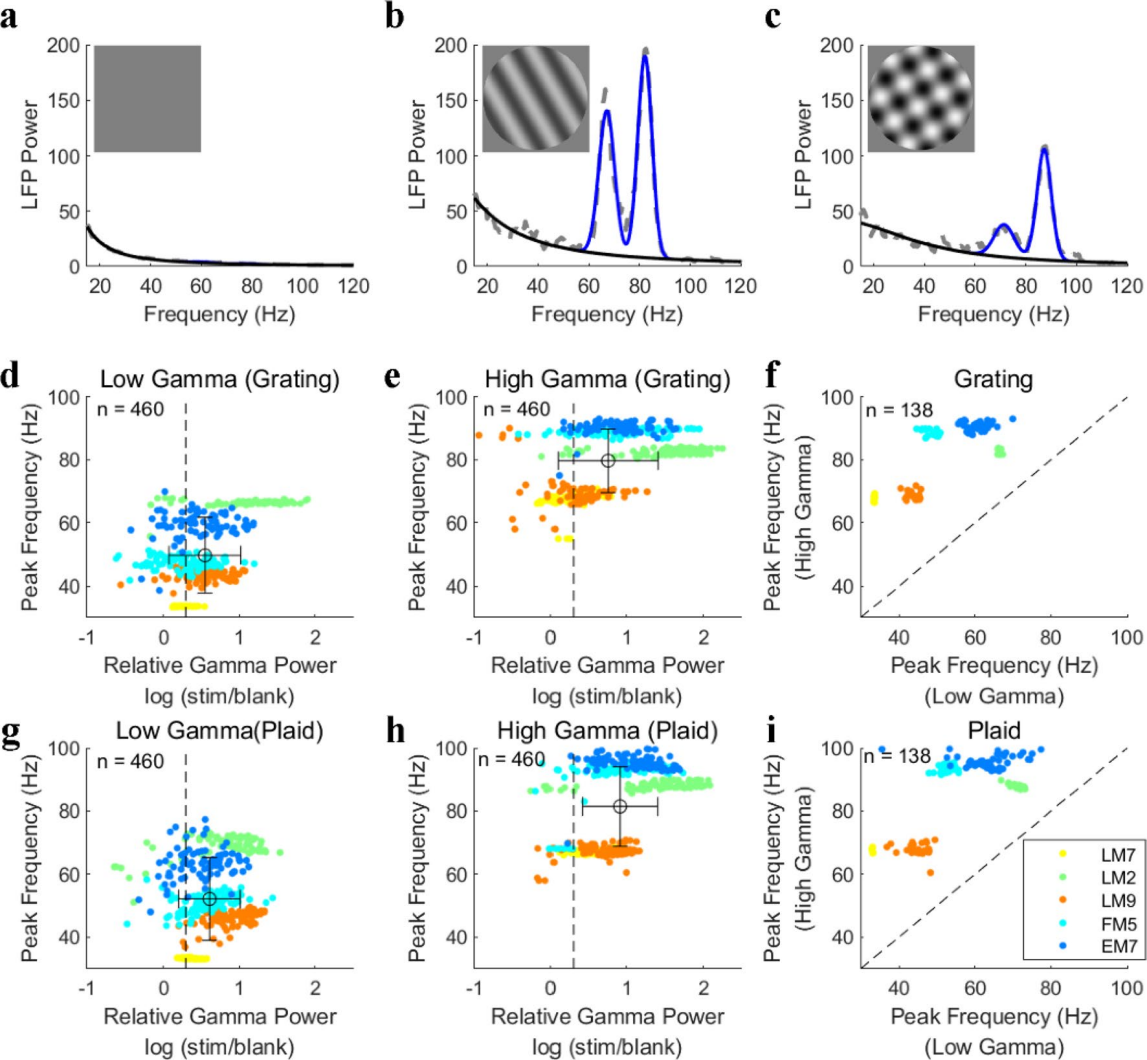
Two distinct gamma oscillations are induced by drift gratings and plaids. Subfigures (**a**), (**b**) and (**c**) demonstrate LFP power spectrums induced by a blank condition (**a**), grating (**b**), and plaid (**c**) respectively. The size and spatial frequency of these stimuli were 38° and 0.05 cycle/^o^. In (**a**), (**b**), and (**c**), gray lines indicate the raw LFP spectrums. Blue curves and black curves indicate the estimated narrow-band gamma power and baseline power through the spectrum fitting procedure. (**d**) shows the peak frequency and relative gamma power of the low gamma oscillation (narrow-band) induced by the drifting grating for all the recording sites. The horizontal error bar represents the mean and standard deviation of the relative gamma power and the vertical error bar represents that of peak frequency. Similar to (**d**), (**e**) shows the peak frequency and relative gamma power of the high gamma oscillation (narrow-band) induced by the drifting grating for all the recording sites. (**f**) Presents a comparison between the peak frequency of the low gamma and high gamma oscillations for sites with all signals good (n = 138: LG, HG, and MUA). (**g**)–(**i**), the same as (**d**)–(**f**), but for gamma oscillations induced by a plaid stimulus. Note that the relative gamma power is defined as the ratio between the stimulus-driven gamma power and the baseline. The threshold (black dotted line) for selecting salient gamma oscillations is labeled in subfigures (**a**), (**e**), (**g**), and (**h**).

Strong MUA and LFP responses were activated after the onset of stimuli and remained relatively high amplitude compared with those under the blank condition (Fig. 1b,c). Apart from the transient response, sustained oscillatory components were observed in the LFP during the later stimulus presentation periods (0.3–2 s). Interestingly, two salient narrow-band gamma oscillations were found in the visual cortex of all five animals under stimulus conditions (at high contrast; see Fig. 1d for an example site and Fig. 2 for population results). A spectrum fitting procedure (detailed in “Materials and methods”) was utilized to estimate the gamma components in the LFPs to capture the narrow-band gamma oscillation (Fig. 2). This fitting procedure^35^ had an excellent performance for explaining the power spectrums under all conditions (the average goodness of fit was 0.938 ± 0.028). We defined the two narrow-band gamma oscillations (blue curves in Fig. 2b,c) as low gamma (LG) and high gamma (HG) based on their relative frequency range and the broad-band component as baseline power (black curves in Fig. 2b,c).

To confirm the generality of the coexistence of two distinct gamma oscillations, we calculated the LFP spectrums of all 480 recording sites. The gamma components for most recording sites (460/480) can be well captured by the Spectrum Fitting method, and 53% of the recorded sites (254/480) showed two distinct gamma oscillations. These gamma oscillations were induced by drift grating and at least twice as large as their baseline power (Fig. 2d,e). For the later sections, only the sites that had low/high gamma oscillations and MUA responses (n = 138) were selected for further analysis. Interestingly, there was an individual difference for peak frequency of low and high gamma among animals (Fig. 2f,i). The peak frequencies of high gamma or low gamma from different electrodes were highly consistent under different stimulus conditions in the same animal, but peak frequencies for high/low gamma were rather variable among different animals (see five clusters in Fig. 2f,i). However, the cross-animal variability doesn’t affect the definition for low and high gamma oscillations and we put low gamma from individual animals together and term them as low gamma for the rest of the results. Results for high gamma were also presented in this way. Moreover, the plaid also induced two distinct narrow-band oscillations for the majority (73%, 348/480) of the recording sites (Fig. 2g–i). Overall, we found that two distinct narrow-band gamma oscillations coexisted in the cat visual cortex.

### Gamma oscillations are mostly suppressed by an additional grating

For recording sites with all signals good (n = 138), low gamma power induced by the drifting grating at high contrast (45%) was significantly suppressed (p < 0.001, Figure S1a) when the orthogonal grating (contrast = 45%) was added (Table S1). However, on average, MUA was significantly enhanced (p < 0.001, Figure S1b) for superimposed gratings, and there was no significant difference (p = 0.23, Figure S1c) for high gamma power induced by single gratings and superimposed gratings (Table S1). We also got similar results with more data points by using qualified sites for each of three signals (LG, n = 325; HG, n = 375; MUA, n = 183) (Figure S1d–f). These results imply that superimposed gratings had different modulation on low gamma, high gamma, and MUA.

**Figure 3:**
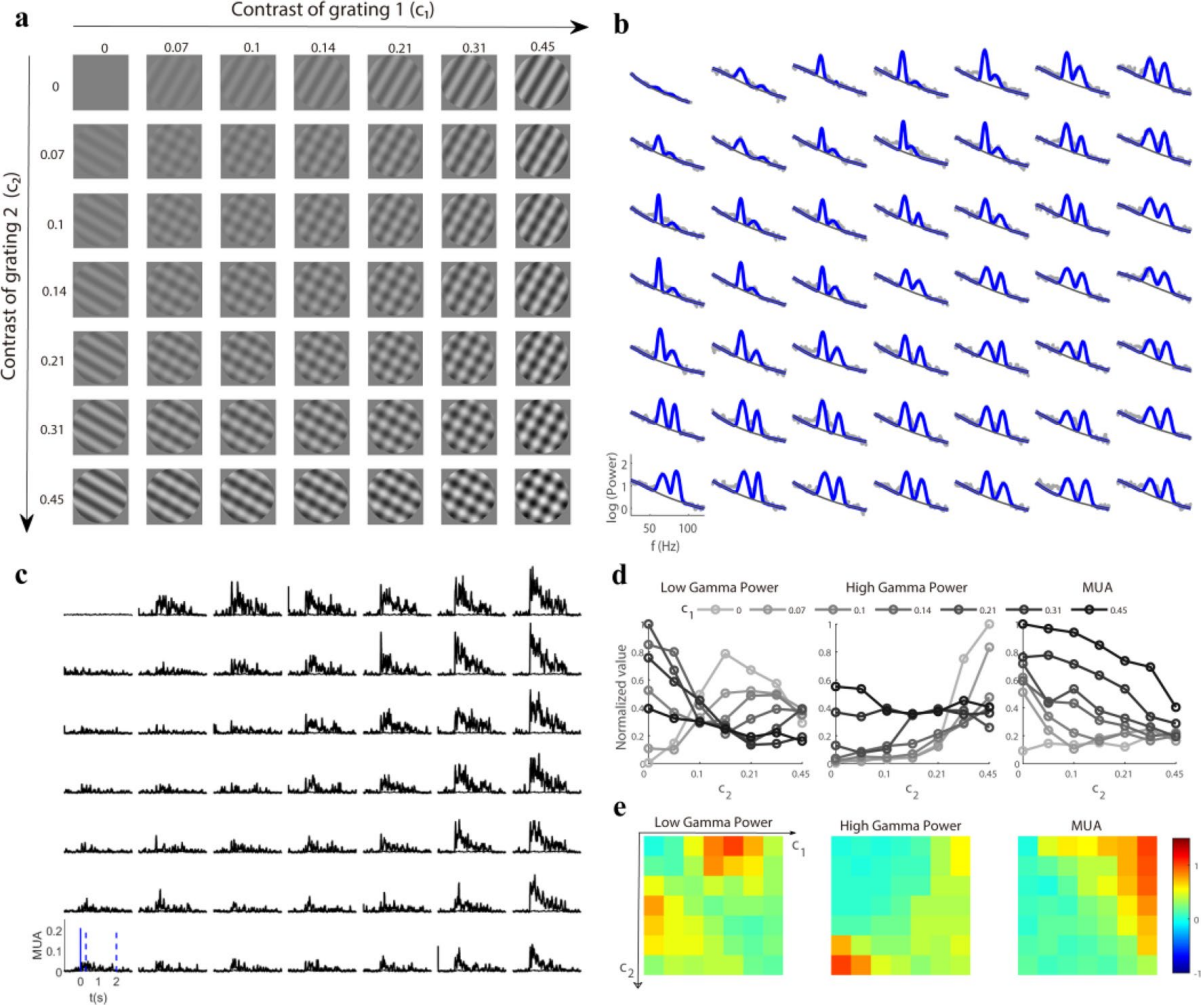
Response matrix was driven by plaid for an example site. (**a**) presents various plaids formed by the linear summation of two orthogonal drift gratings with varying contrast. These stimuli (size, 38°; spatial frequency, 0.05 cycle/^o^) were presented for 2 s with a time–frequency of 2 Hz and were repeated 10 times. (**b**) shows the trial averaged (n = 10) LFP power spectrum (gray dots) in response to corresponding stimuli in a. Two narrow-band gamma oscillations (blue curve) and the baseline (dark gray line) were estimated through a spectrum fitting procedure. (**c**) shows trial averaged (n = 10) spiking activity (MUA) driven by the stimuli in (**a**). The appearance of stimuli is labeled by a solid blue line in (**c**). The time-averaged (time period marked by the two dotted lines) MUA was used for (**d**) and (**e**). (**d**) presents several contrast (*c*_2_) tuning curves with the contrast of grating 1 (*c*_1_) fixed for low gamma power, high gamma power, and MUA. (**e**) demonstrates the response matrices of low gamma power, high gamma power, and spiking activity (MUA) in response to stimuli in (**a**). Note that all the responses in (**d**) and (**e**) were normalized by their maximum value.

To get a further understanding of how these three signals (LG, HG, and MUA) change with superimposed gratings, we measured spiking activity and gamma oscillations activated by various plaids. Plaids were formed by the summation of two orthogonal drift gratings with independently varying contrast (*c*_1_, *c*_2_). Both gratings had seven contrast levels, allowing 49 plaids to be attained (Fig. 3a). LFP spectrums were calculated for each plaid during the sustained time course (Fig. 3b), and the corresponding MUA responses were also recorded (Fig. 3c). The stimulus effects on gamma oscillations were investigated by fixing the contrast (*c*_1_) of the base grating and obtaining the contrast (*c*_2_) tuning of the mask grating for LG and HG. The gamma power was enhanced with increasing contrast (*c*_2_) when only the drift grating was presented (*c*_1_ = 0), whereas an additional grating (*c*_1_ > 0) led to the suppression of gamma power (Fig. 3d). The responses for the superimposed gratings were then calculated as a response matrix for gamma oscillations and MUA (Fig. 3e).

**Figure 4:**
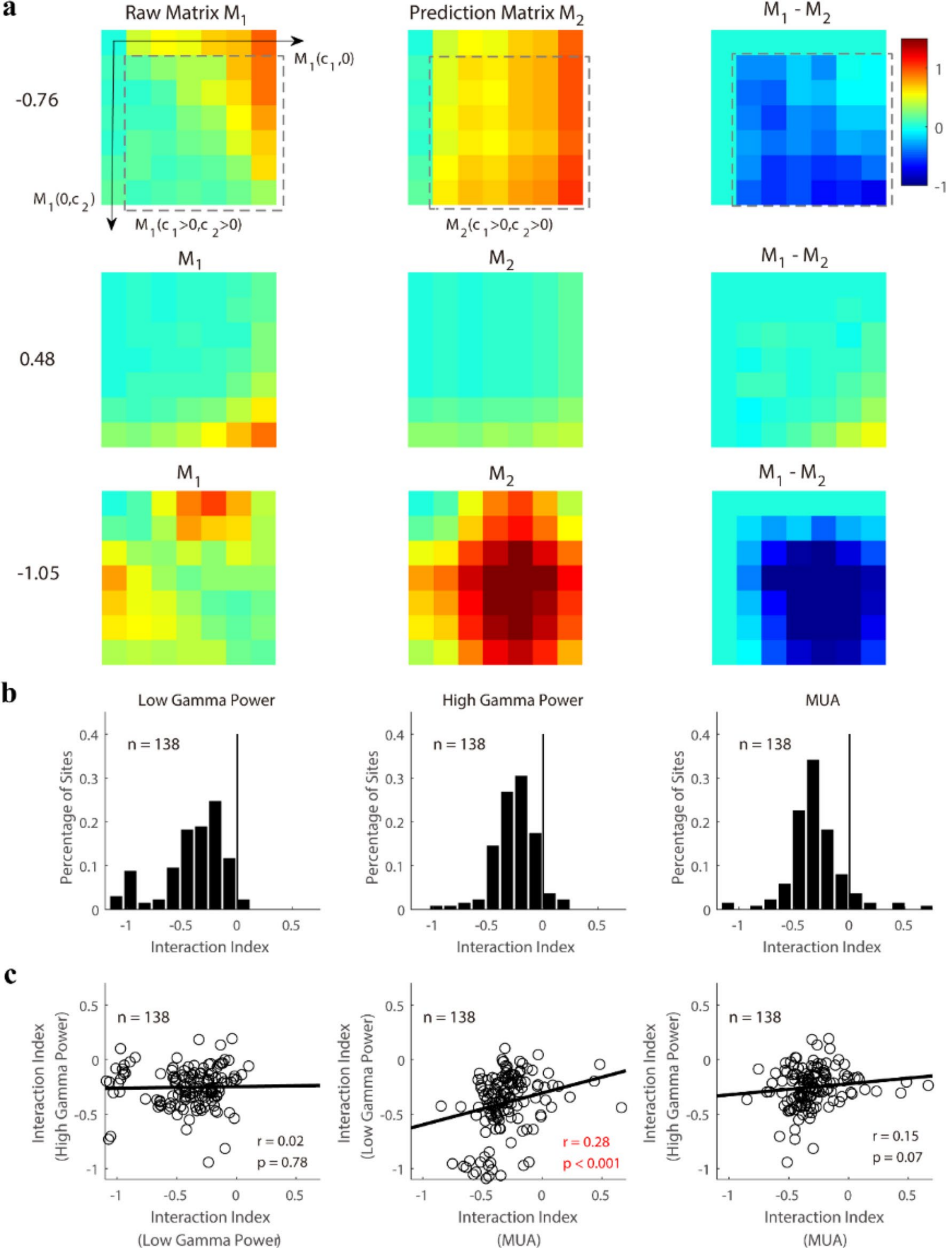
The interaction indices measured from response patterns of gamma oscillations and MUA. (**a**) demonstrates the response matrices (M_1_) in response to stimuli in Fig. 3a for three example sites. The prediction matrix (M_2_) can be calculated as: M_2_(*c*_1_, *c*_2_) = M_1_(*c*_1_, 0) + M_1_(0, *c*_2_). The interaction index was then defined by the Eq. (6) in “Materials and methods”. The values of the interaction index were labeled by the side of each example. (**b**) shows histograms of the interaction indices for low gamma power (LG), high gamma power (HG), and MUA. The index of most sites (n = 136 for LG; n = 132 for HG; n = 128 for MUA) was significantly lower (Bootstrap method, p < 0.001) than 0 (black solid line). (**c**) Shows the site by site correlations of interaction indices among three signals (from left to right, they are the correlation between LG and HG, the correlation between LG and MUA, and the correlation between HG and MUA). Pearson’s correlation was used to test the significance of the relationship in each pair of comparisons (significant correlation was marked as a red font). Linear regression (black lines in **c**) was also calculated for correlation measurements.

To quantify the interaction effect of the two superimposed gratings, we defined an interaction index as the difference between the prediction matrix (the linear summation of responses induced by two-component gratings) and the raw response matrix (Fig. 4a). If the gamma oscillation was suppressed when the additional grating was added, the interaction index was negative, while a positive interaction index meant facilitation. The index of most sites was significantly lower (p < 0.001) than 0 (n = 136 for LG; n = 132 for HG; n = 128 for MUA in Fig. 4b). This result implies that for the majority of sites, the gamma oscillations and MUA showed cross-orientation suppression^8,29^. Some sites also showed cross-orientation facilitation^32,36^ for both gamma oscillations and MUA (Fig. 4b, n = 2 for LG; n = 6 for HG; n = 10 for MUA). Surprisingly, there was no significant correlation between the interaction index of the low gamma power and high gamma power (r = 0.02, p = 0.78 in Fig. 4c); the same was true for the interaction index of MUA and that of high gamma oscillation (r = 0.15, p = 0.07 in Fig. 4c). MUA and low gamma had weak correlation (r = 0.28, p < 0.001 for LG and MUA) for their interaction indices. Taken together, for most sites, gamma oscillations were suppressed by the additional grating in the plaid stimuli. However, the interaction index of MUA is weakly correlated with that of low gamma and no significant correlation was found between the interaction index of high gamma and that of MUA.

### Response patterns of gamma oscillations are different to those of MUAs

**Figure 5:**
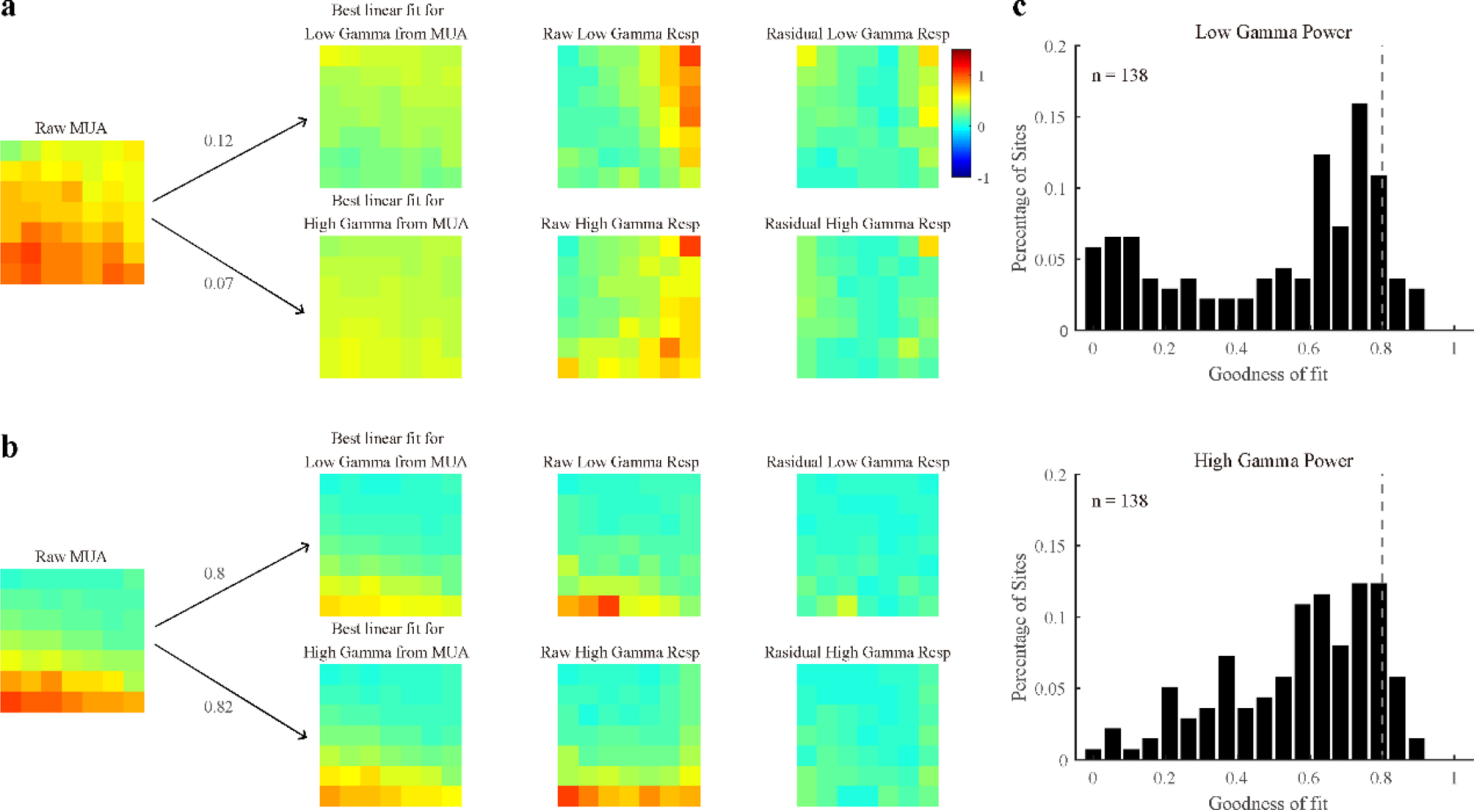
Linear predictions for response patterns of gamma oscillations from those of MUA. Subfigures (**a**) and (**b**) demonstrate the linear predictions and residuals for response patterns of low and high gamma from that of MUA in two example sites. The goodness of fit of these two examples was calculated and labeled in subfigures (**a**) and (**b**). Note that subfigure a represents a poor prediction of the response pattern of gamma oscillations from that of MUA, whereas subfigure b demonstrates a good one. Using the same method, the population goodness of fit of the linear prediction of the response pattern of low gamma (top) and high gamma power (bottom) is presented in (**c**). Note that the goodness of fit threshold (gray dotted line) is labeled in the histogram.

We further checked whether the response patterns of gamma oscillations were similar to the MUA response patterns. Gamma response patterns were estimated through a linear fit from the MUA response matrix (Fig. 5a), allowing the residual and goodness of fit for a single site to be acquired (Fig. 5a,b). The goodness of fit for most sites was less than 0.8 (0.50 ± 0.28, mean and standard deviation for LG; 0.57 ± 0.21 for HG; Fig. 5c). In short, the gamma oscillations and MUA differ not only in their interaction index but also in their response patterns.

### A normalization model for both gamma oscillations and MUA

**Figure 6:**
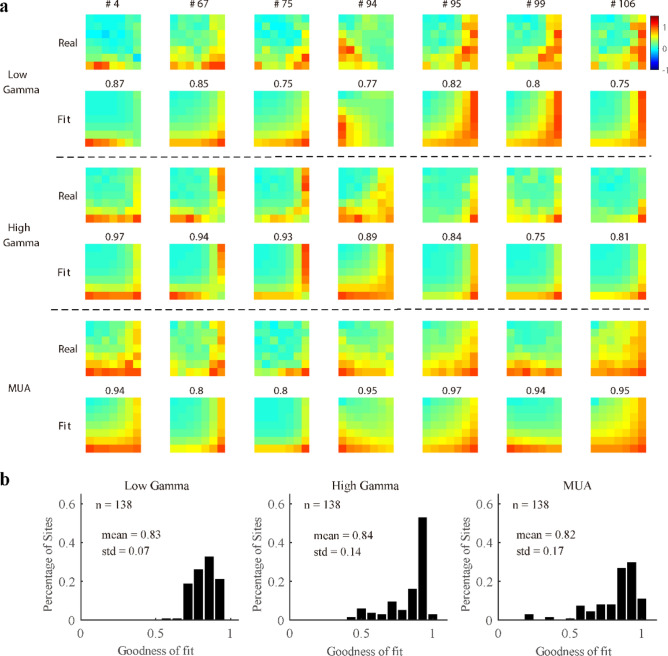
Good performance of the normalization model for response patterns of gamma oscillations and MUA. (**a**) Shows the real response and corresponding fit response of low gamma, high gamma, and MUA for several example sites. The goodness of fit is labeled above each example. (**b**) Presents histograms of the goodness of fit of the normalization model for one data group (138 sites where both gamma oscillations and MUA were all good).

Previously, we demonstrated the differences between the response patterns of gamma oscillations and MUA. Interestingly, they share a common feature known as cross-orientation suppression, which has been explained by applying the normalization model to spiking activity^28,29,31^. To test whether the normalization model can also regulate the response patterns of gamma oscillations, we derived a modified normalization model with the following form (Eqs. 8–10):

8 $${{R}_{1}=R}_{max}\cdot \frac{{c}_{1}^{{m}_{1}}}{{{c}_{50}}^{{m}_{2}}+{({c}_{1}+{c}_{2})}^{{m}_{2}}},$$

9 $${{R}_{2}=R}_{max}\cdot \frac{{c}_{2}^{{m}_{1}}}{{{c}_{50}}^{{m}_{2}}+{({c}_{1}+{c}_{2})}^{{m}_{2}}},$$

10 $$R=b\cdot {R}_{1}+\left(1-b\right)\cdot {R}_{2}+{R}_{0},$$

where, R_1_ and R_2_ in Eqs. (8) and (9) are the normalization process in the visual system, c_50_ is half-maximal contrast, m_1_ and m_2_ are constant exponents, and R_max_ estimates the maximum stimulus-driven responses. The recorded response is defined as the weighted sum of R_1_ and R_2_, and parameter b denotes the weight. Note that c_50_, m_1_, and m_2_ are related to the process of normalization, while b is related to the orientation tuning of the individual site. The normalization model accounted well for low gamma, high gamma, and MUA (Fig. 6a). For the data where all three signals were good (n = 138), the mean values of the goodness of fit were 0.83 ± 0.07 for LG, 0.84 ± 0.14 for HG, and 0.82 ± 0.17 for MUA (Fig. 6b). For data where a single signal was good (n = 325 for LG; n = 375 for HG; n = 183 for MUA), the mean values of the goodness of fit were also high (Figure S5a; 0.8 ± 0.09 for LG, 0.81 ± 0.16 for HG, and 0.81 ± 0.17 for MUA).

**Figure 7:**
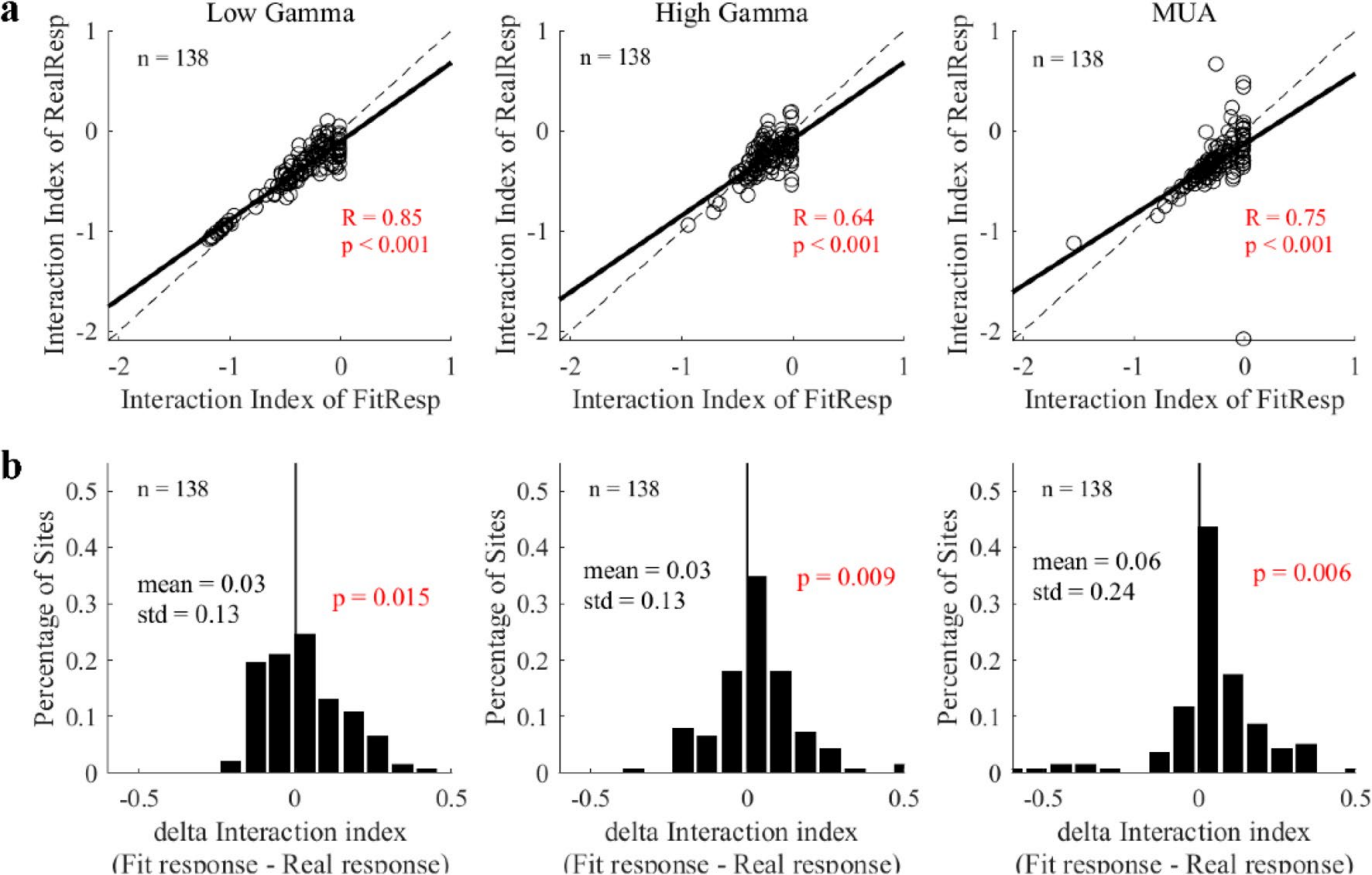
Strong correlations between the interaction index of fit responses and that of real responses. (**a**) Shows comparisons between the interaction index of real responses and that of the fit responses for low gamma, high gamma, and MUA within a data group (138 sites where both gamma oscillations and MUA were all good). Linear regression was calculated to confirm the linear correlation (the black solid line). (**b**) Presents histograms of the differences between the fit responses and real responses. Note that Pearson’s correlation was used to test the significance of the relationship between the two indices, and the bootstrap method was used to test whether the distribution of the delta interaction index was significantly different from 0 (black line in subfigure **b**). All the significant correlations were labeled as red fonts.

Furthermore, for sites with all signals good (n = 138), the interaction index of the model was also comparable with those of the real responses for low gamma, high gamma and MUA (LG, r = 0.93, p < 0.001; HG, r = 0.72, p < 0.001; MUA, r = 0.72, p < 0.001; Fig. 7a). There was a significant difference among interaction index from different signals (p = 0.002 for LG; mean difference = 0.03, p = 0.009 for HG; p = 0.006 for MUA; Fig. 7b), but the difference was very small and negligible (mean difference = 0.03 for LG, mean difference = 0.03 for HG, mean difference = 0.06 for MUA). Besides, the interaction index of fit response and that of real responses were significantly correlated (LG, r = 0.84, p < 0.001; HG, r = 0.48, p < 0.001; MUA, r = 0.54, p < 0.001; Figure S5b in supplementary information) for large data groups (LG, n = 325 sites, HG, n = 375 sites, MUA, n = 183 sites). There was only small difference between the interaction index for fit and real responses (mean difference = 0.02, p = 0.13 for LG; mean difference = 0.03, p < 0.001 for HG; mean difference = 0.07, p < 0.001 for MUA; Figure S5c). Taken together, both gamma oscillations and MUA can be explained by the same form of normalization model.

### Response patterns of gamma oscillations to plaids are more diverse than those of MUAs

**Figure 8:**
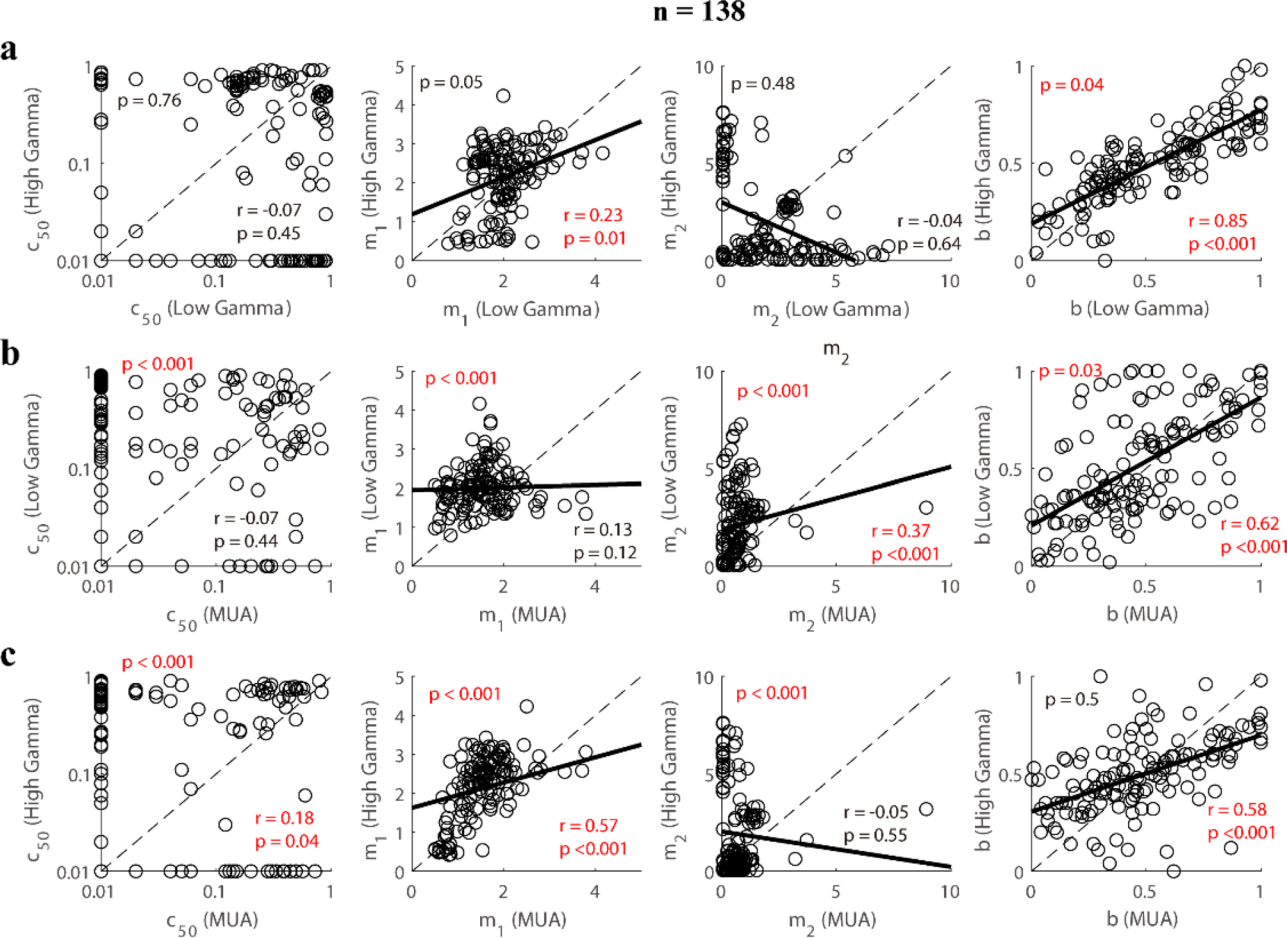
Comparisons of fitting parameters in the normalization model among three signals (low gamma, high gamma, and MUA). (**a**) shows the correlations between the fitting parameters (*c*_50_, *m*_1_, *m*_2_, *b*) of low gamma and high gamma. Similar to (**a**), the correlations between the fitting parameters (*c*_50_, *m*_1_, *m*_2_, *b*) of low gamma and MUA are shown in (**b**). (**c**) presents the correlations between the fitting parameters (*c*_50_, *m*_1_, *m*_2_, *b*) of high gamma and MUA. Note that Pearson’s correlation (p-value in the upper left corner) was used to test the significance of each correlation. The bootstrap method (p-value in the lower right corner) was used to test whether the scatter diagrams were significantly biased from the diagonal line (black dotted line). Linear regression was also calculated to confirm the linear correlation for each pair of variables (the black solid line). All the significant statistical analyses were labeled as red fonts.

In the previous section, we showed that the response patterns of gamma oscillations were different from those of MUA. The normalization model gives us the opportunity to see in what aspects the gamma and MUA differ. We compared the fitting parameters (*c*_50_, *m*_1_, *m*_2_, and *b*) of the two gamma oscillations and MUA. There were no significant correlations for parameter c_50_ among the 3 signals (r = 0.07, p = 0.41 for LG and HG; r = − 0.01, p = 0.93 for LG and MUA; r = 0.15, p = 0.07 for HG and MUA), while there existed significant correlations for parameter *b* among the three signals (r = 0.82, p < 0.001 for LG and HG; r = 0.63, p < 0.001 for LG and MUA; r = 0.53, p < 0.001 for HG and MUA; Fig. 8). The parameter m_1_ for low gamma was weakly correlated with that for high gamma (r = 0.32, p < 0.001) and not significantly correlated with that for MUA (r = 0.05, p = 0.53). And the parameter m_1_ for high gamma was weak correlated with that for MUA (r = 0.37, p < 0.001). For parameter m_2_, correlation between low gamma and high gamma (r = − 0.25, p < 0.001) was significant, but the correlation between low gamma and MUA (r = 0.16, p = 0.06) or between high gamma and MUA (r = − 0.07, p = 0.4) was not significant. These results indicate different mechanisms behind the response patterns of the gamma oscillations and MUA to superimposed gratings.

**Figure 9:**
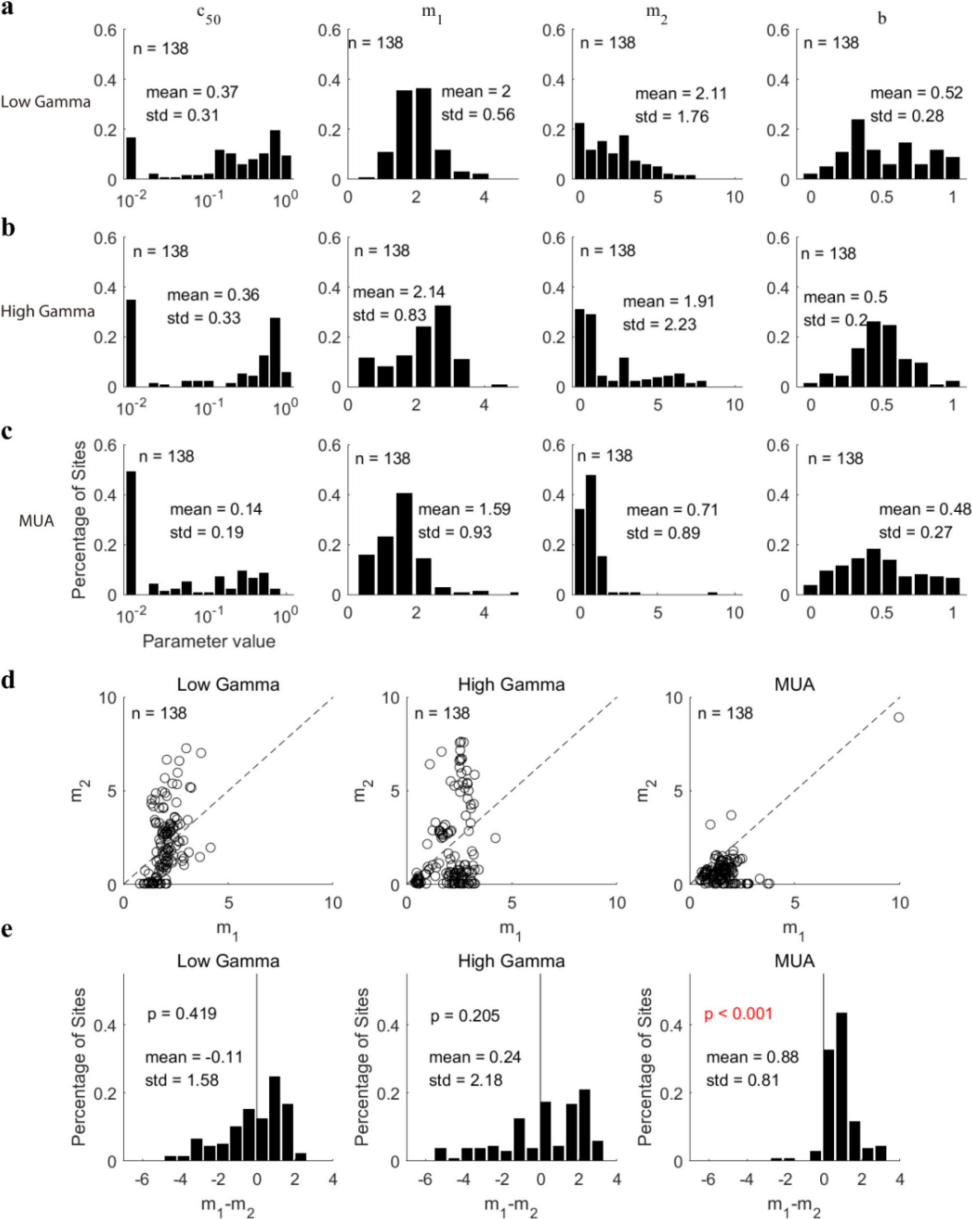
Analysis of the fitting parameters in the normalization model for sites with all signals good (low gamma, high gamma, and MUA, n = 138). (**a**) Shows histograms of various fitting parameters (c_50_, m_1_, m_2_, (**b**) for low gamma. Subfigures (**b**) and (**c**) are similar to (**a**), but for high gamma and MUA respectively. (**d**) presents scatter diagrams of the parameters m_1_ and m_2_ for three signals. (**e**) shows histograms of the difference between m_1_ and m_2_ for these three signals. The bootstrap method was used to test whether the distributions were significantly biased from 0 (black solid line). All the significant correlations were labeled as red fonts.

To obtain further insight into the difference between the response patterns of gamma oscillations and those of MUA, population fitting parameters for the three signals were compared. There was no significant difference (p = 0.1 for LG and MUA; p = 0.31 for HG and MUA; p = 0.16 for LG and HG) between gamma oscillations and MUA for parameter b (0.52 ± 0.28 for LG; 0.5 ± 0.2 for HG; 0.48 ± 0.27 for MUA; Fig. 9a–c). However, there were significant differences (p < 0.001 for LG and MUA; p < 0.001 for HG and MUA) between the gamma oscillations and MUA for other fitting parameters (c_50_, m_1_, and m_2_), with gamma oscillations having higher mean values and variance than MUA (For LG, c_50_: 0.37 ± 0.31, m_1_: 2 ± 0.56, m_2_: 2.11 ± 1.76, Fig. 9a; For HG, c_50_: 0.36 ± 0.33, m_1_: 2.14 ± 0.83, m_2_: 1.91 ± 2.23, Fig. 9b; For MUA, c_50_: 0.14 ± 0.19, m_1_: 1.59 ± 0.93, m_2_: 0.71 ± 0.89; Fig. 9c). Note that the major differences between gamma oscillations and MUA were caused by fitting parameters m_1_ and m_2_. We then compared m_1_ and m_2_ site-by-site in the two gamma oscillations and MUA (Fig. 9d). Many sites had m_2_ higher than m_1_ for both high gamma oscillations and low gamma oscillations, but m_2_ is lower than m_1_ for MUA from almost all sites (p < 0.001). The fitting parameter domain (m_1_–m_2_) of low gamma and high gamma was more widely distributed than MUA (Fig. 9e). The diversity in fitting parameters for gamma oscillations is mainly because m_1_–m_2_ of low gamma and high gamma for many recording sites were smaller than 0 (44% recording sites for high gamma; 35% recording sites for low gamma), but m_1_–m_2_ was higher than 0 for most recording sites of MUA (95% recording sites) (see consistent results in figure S6 for data where a single signal was good, n = 325 for LG; n = 375 for HG; n = 183 for MUA).

### Relationship between model parameters and sites’ response properties

**Figure 10:**
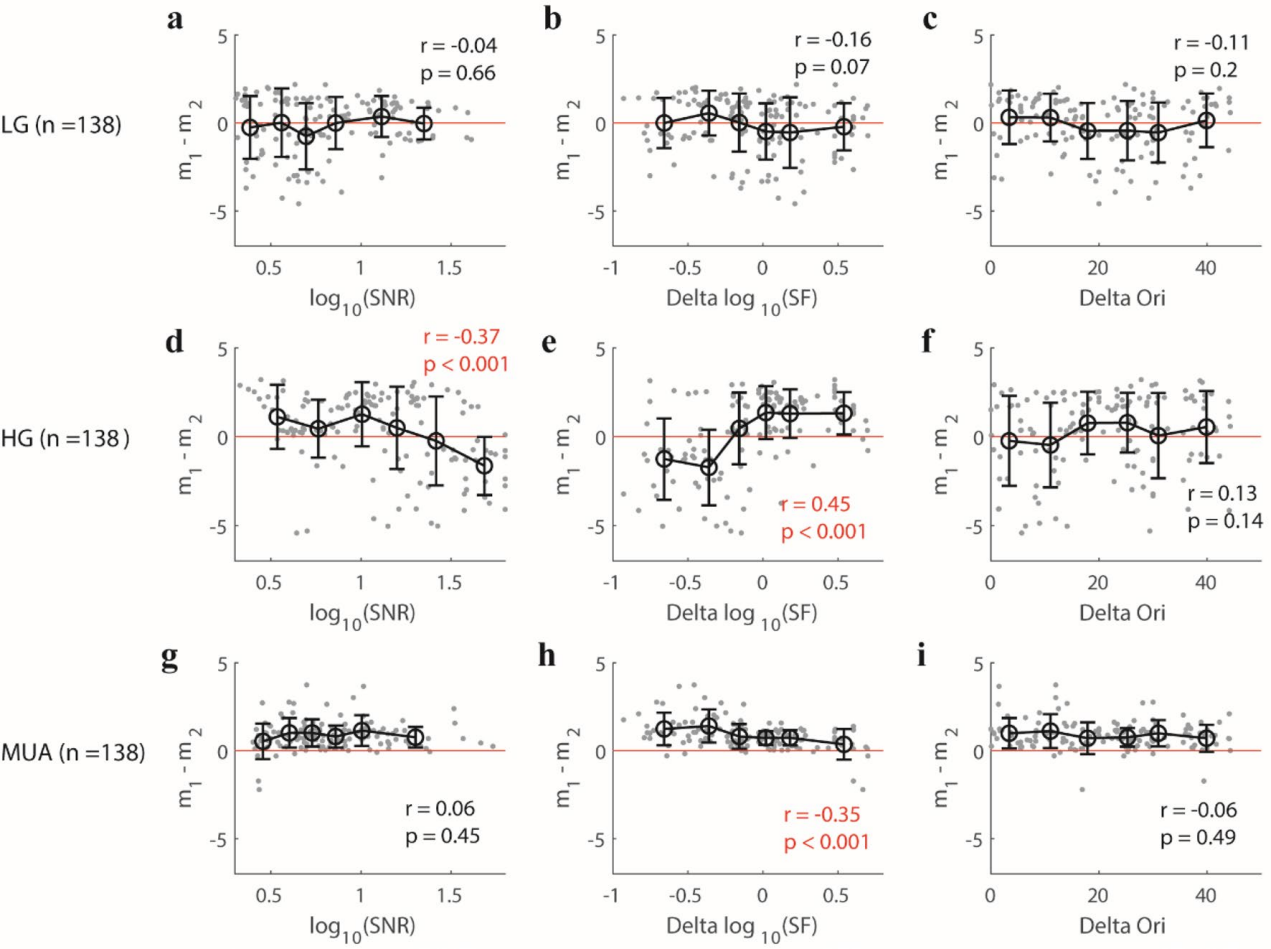
The relationship between several factors (SNR, Delta SF, and Delta Ori) and fitting parameter (m_1_–m_2_) for three signals (low gamma, high gamma, and MUA). For low gamma (LG, n = 138), (**a**) shows the scatter plot of signal–noise ratio (SNR) and (m_1_–m_2_); (**b**) presents scatter plot of the Delta SF (the ratio of the chosen stimulus spatial frequency and preferred spatial frequency of individual sites) and m_1_–m_2_; (**c**) shows the scatter plot of Delta Ori (the difference between the chosen stimulus orientation and preferred orientation of individual sites) and (m_1_–m_2_). The data points in subfigures (**a**–**c**) were divided into six groups based on their ranking order for values of each variable. The error bar in each group denotes the mean value (black circle) and standard deviation (black bar) of (m_1_–m_2_). Subfigures (**d**–**i**) are similar to (**a**–**c**), but (**d**–**f**) are for high gamma (HG, n = 138), while, (**g**–**i**) are for MUA (n = 138). Note that Spearman’s correlation was used to check their relationship. All the significant correlations were labeled as red fonts.

In the previous section, we found that parameters (m_1_–m_2_, m_1_, m_2_) in the normalization model for gamma oscillations were different from those model parameters for MUA. Two possibilities might explain such parameter differences for gamma. One possibility is that parameters (m_1_–m_2_) is mainly determined by noise level of a signal, and the noise level of gamma oscillations might be more variable than those of MUA due to either recording properties or response properties for individual sites. If this possibility is true, then we should expect that (m_1_–m_2_) for the three different brain signals (HG, LG, and MUA) are all correlated with their signal–noise level (SNR) in a similar way, and sites with high SNRs will have similar (m_1_–m_2_) for all three brain signals. The other possibility is that parameters (m_1_–m_2_) in the normalization model are mainly determined by neural circuitry underlying each of the three signals. If the second possibility is true, then we might also see a correlation between SNRs and (m_1_–m_2_) for the three signals but in different correlation trends due to their different underlying mechanisms. In the experiment, we used a single set of stimulus parameters (two orthogonal stimulus orientations and one spatial frequency) to test a large population of simultaneously recorded neurons with diverse tuning properties (different orientation preferences and spatial frequency preferences). The mismatch of stimulus parameters and some sites’ preferences for orientation or spatial frequency will activate neural mechanisms at different levels and lead to different relative noise levels for the three brain signals at different recording channels. To test the above two possibilities, we correlated m_1_–m_2_ with three factors, SNR of the three signals, the ratio between the stimulus spatial frequency and preferred spatial frequency for individual sites (Delta SF), and the difference between the stimulus orientation and preferred orientation for individual sites (Delta Ori). To capture the trend of m_1_–m_2_ with different factors, we divided our data into six groups, each of which had a similar number of recording sites based on the factor’s value order, and calculated the mean and standard deviation for m_1_–m_2_ in each group (as shown in Fig. 10).

For low gamma, no significant correlation was found between m_1_–m_2_ and three factors (SNR: r = − 0.04, p = 0.66, Fig. 10a; Delta SF: r  = − 0.16, p = 0.07, Fig. 10b; Delta Ori: r = − 0.11, p = 0.2, Fig. 10c). More importantly, there always exist some recording sites with (m_1_–m_2_) less than 0 in all bins of SNR, Delta SF, and Delta Ori, which suggests that the three factors do not strongly affect the m_1_–m_2_ value for low gamma.

Interestingly, the relationship between m_1_–m_2_ and the three factors for high gamma oscillations was rather different from that for low gamma. For high gamma, m_1_–m_2_ was significantly correlated with SNR and Delta SF (r = − 0.37, p < 0.001, for SNR; r = 0.45, p < 0.001, for Delta SF), and no significant correlation was found between m_1_–m_2_ and Delta Ori (r = 0.13, p = 0.14). The recording sites with negative m_1_–m_2_ mostly had high SNR (Fig. 10d). More interestingly, m_1_–m_2_ was clearly related to Delta SF (Fig. 10e). When the chosen SF for visual stimulus was lower than a site’s preferred SF (Delta SF < 0 in Fig. 10e), more sites had negative m_1_–m_2_, but when Delta SF was larger than 0 (chosen SF for visual stimulus was higher than a site’s preferred SF), more sites had positive m_1_–m_2_ (Fig. 10e; also see consistent results in figure S7 for data where a single signal was good, n = 325 for LG; n = 375 for HG; n = 183 for MUA).

Overall, besides the relationship between m_1_–m_2_ and the three factors, negative (m_1_–m_2_) existed in most bins of different factors for gamma oscillations (except in the bins with low Delta SF). On the contrary, m_1_–m_2_ for MUA was higher than 0 for most recording sites under all conditions (Fig. 10g–i). Interestingly, there also existed a negative correlation between m_1_–m_2_ and Delta SF (r = − 0.35, p < 0.001) for MUA. Taken together, m_1_–m_2_ of gamma oscillations are more diverse than that of MUA under all factors. What’s more, this diversity for high gamma is mainly related to the specific spatial frequency (a site’s m_1_–m_2_ is likely to be negative when stimulus SF is lower than the site’s SF preference) but less related to a specific orientation. The different relationship between (m_1_–m_2_) and the three factors for LG, HG, and MUA rules out the possibility that model parameters are mainly determined by the noise level of recording signals (possibility one), but support the idea that normalization of the three brain signals is due to different neural mechanisms.

**Figure 11:**
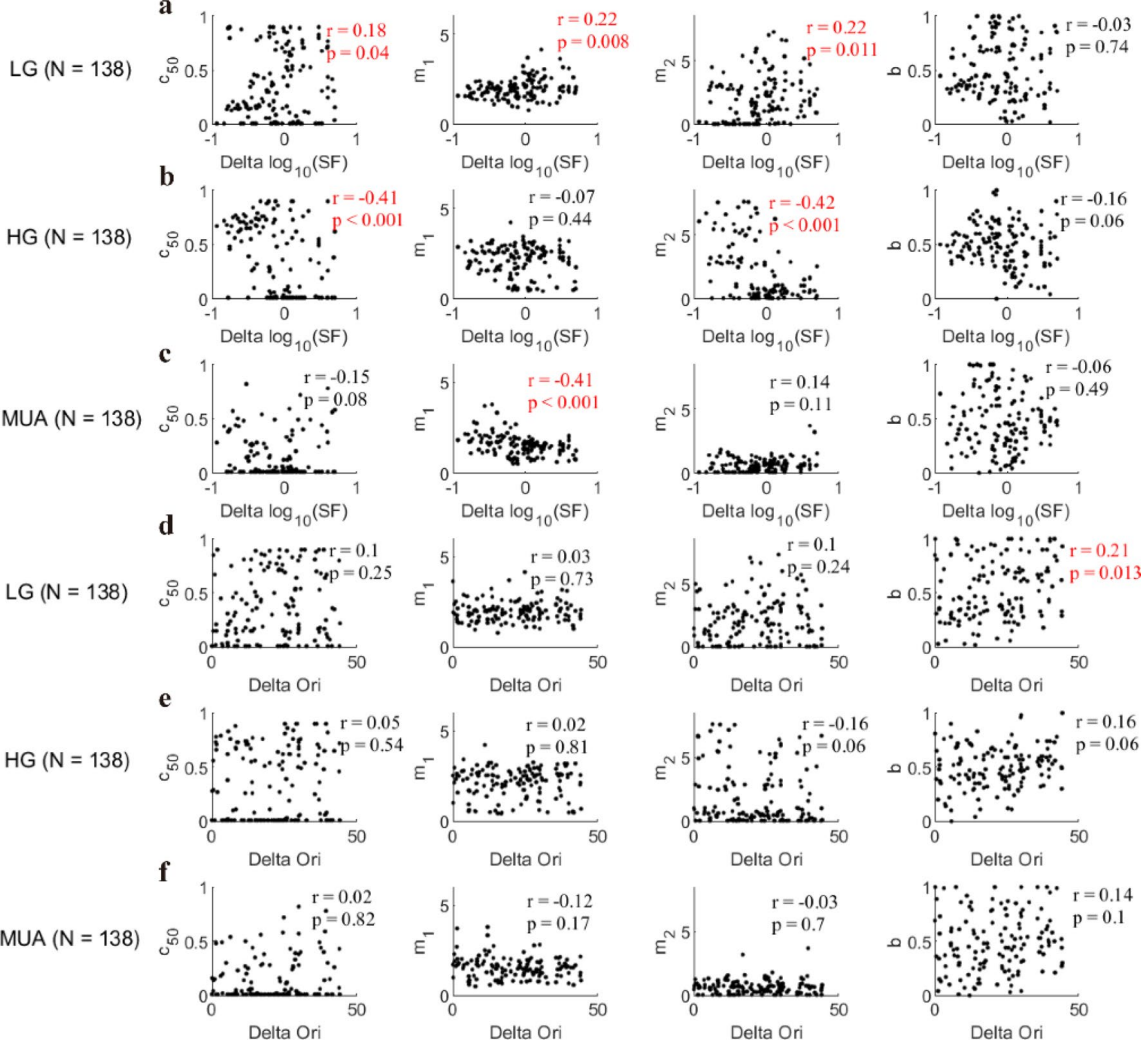
The relationship between fitting parameters and stimulus factors (Delta SF and Delta Ori). Subfigure (**a**) presents the relationship between Delta SF and the four fitting parameters (from left to right: c_50_, m_1_, m_2_, (**b**) for low gamma (LG, n = 138 sites). Subfigures (**b**) and (**c**) are similar to (**a**), but for high gamma (HG, n = 138 sites) and MUA (n = 138) respectively. Subfigure (**d**) shows the scatter plot of Delta Ori and the four fitting parameters (from left to right: c_50_, m_1_, m_2_, (**b**). Note that Spearman’s correlation was used to account for the relationship between the stimulus factors and the fitting parameters. Subfigures (**e**) and (**f**) are similar to (**d**), but for high gamma and MUA respectively. All the significant correlations were labeled as red fonts.

To get a more comprehensive understanding of model parameters m_1_ and m_2_ as well as (m_1_–m_2_), we correlated all the four model parameters (c_50_, m_1_, m_2_, and b) with Delta SF and Delta Ori. Compared with previous results for Delta SF and (m_1_–m_2_) (Fig. 10e,h), the correlation between m_1_–m_2_ and Delta SF for high gamma was determined by m_2_, but that for MUA was mainly determined by m_1_: Normalization parameter m_2_ was correlated with Delta SF for high gamma (m_1_: r = − 0.07, p = 0.44, m_2_: r = − 0.42, p < 0.001; Fig. 11b), while m_1_ was correlated with Delta SF for MUA (m_1_: r = − 0.41, p < 0.001, m_2_: r = 0.14, p = 0.11; Fig. 11c). The insignificant correlation results for low gamma (Fig. 10b) can be explained as the balance between m_1_ and m_2_ (m_1_: R = 0.22, p = 0.008; m2: r = 0.22, p = 0.011; Fig. 11a). Consistent with correlation result for Delta Ori and (m_1_–m_2_) (Fig. 10c,f,i), no significant correlation was found between Delta Ori and m_1_ or m_2_ for all three signals: low gamma (m_1_: r = 0.03, p = 0.73, m_2_: r = 0.1, p = 0.24; Fig. 11d), high gamma (m_1_: r = 0.02, p = 0.81, m_2_: r = − 0.16, p = 0.06; Fig. 11e) and MUA (m_1_: r = − 0.12, p = 0.17, m_2_: r = − 0.03, p = 0.7; Fig. 11f). Parameter c_50_ had correlation with Delta SF for two signals (r = 0.18, p = 0.04 for low gamma; r = − 0.41, p < 0.001 for high gamma; Fig. 11a,b), but no significant correlations were found between Delta SF and b for three signals (r = − 0.03, p = 0.74 for low gamma; r = − 0.16, p = 0.06 for high gamma; r = − 0.06, p = 0.49 for MUA; Fig. 11a–c). Similar results can be found with more data points by using qualified sites for each of three signals (LG, n = 325; HG, n = 375; MUA, n = 183) (Figure S8).

It is worth noticing that model parameter m_2_ has a significant correlation with Delta SF and it has a range wider than that of parameter m_1_ for HG (Fig. 11b), which implies that SF-related m_2_ is the main factor regulating the diversity of normalization for HG. However, parameter m_1_ is more correlated to Delta SF than parameter m_2_ is for MUA (Fig. 11a,c), which suggests that SF-related m_1_ is the main factor regulating the diversity of normalization for MUA. Interestingly, parameter m_1_ had similar trends with m_2_ for LG. Taken together, these correlation results further suggest that low gamma, high gamma, and MUA are regulated by the different manner of normalization; and the relationship between stimulus parameters and model parameters (m_1_, m_2_, and m_1_–m_2_) consistently shows that normalization in high gamma is related to sites’ tunings to spatial frequency.

### The comparison of model parameters between A17 and A18

**Figure 12:**
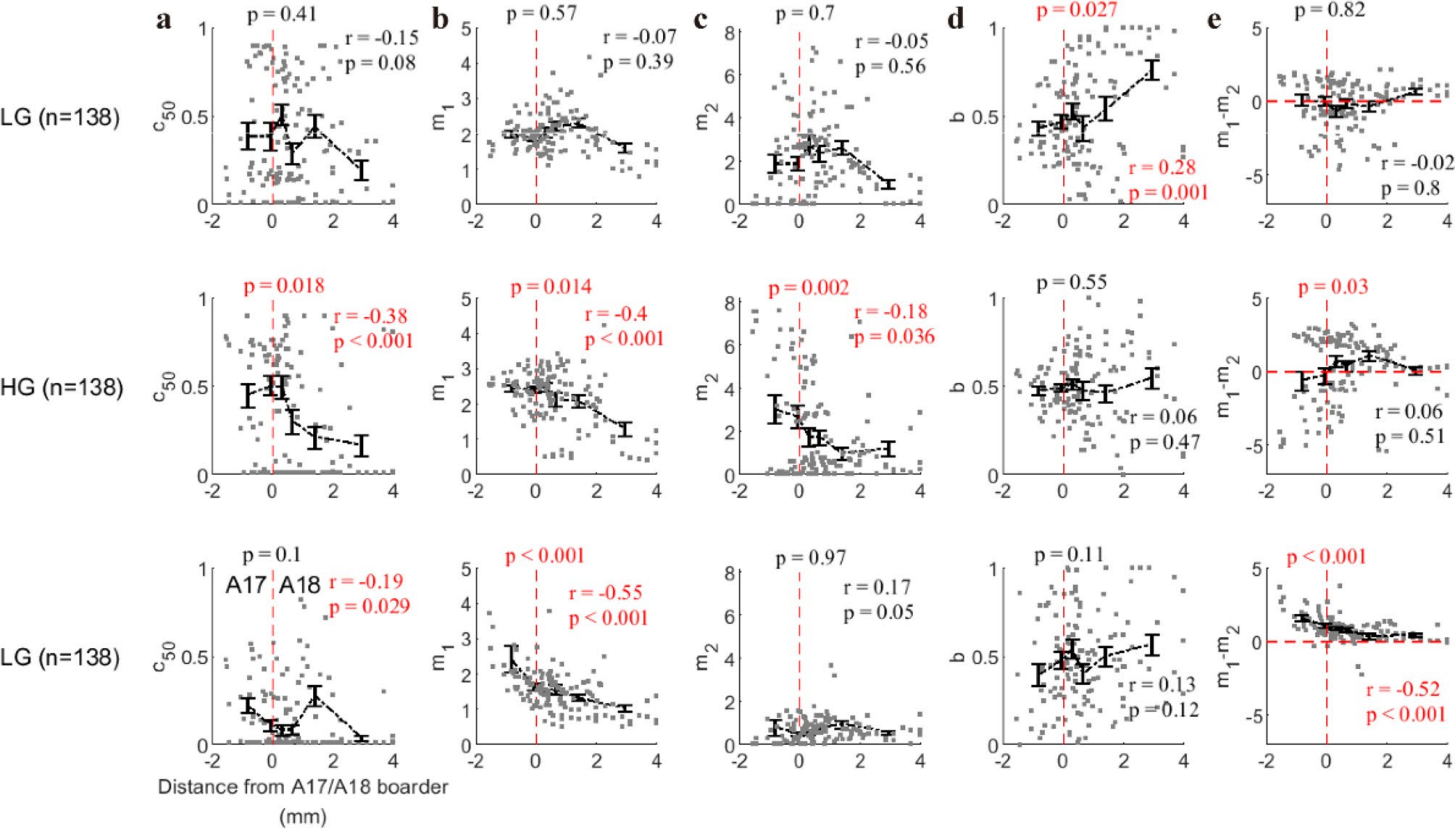
The correlation between brain areas (A17 vs. A18) and fitting parameters. The distance from the border between Area 17 and Area 18 was used to represent the spatial position of the individual recording site. The A17/A18 border was determined through the previous work^38^. Subfigure a shows the relationship between the distance from A17/A18 border (distance index) and fitting parameter c50 for three signals (from top to bottom: low gamma, high gamma, and MUA). Subfigures (**b**–**d**) are similar to (**a**), but they account for the relationship between distance index and fitting parameters m_1_, m_2_, b, and m_1_–m_2_ respectively. Spearman’s correlation was used to account for correlation measurement. Note that the sites with negative distance index are located in A17, while the positive distance index means A18 (zero is marked by red dot line). The p-values (T-test) located above red dashed lines in each subplot represent the statistical significances for whether parameters in brain Area 17 are significantly different from Area 18. The data points in each subfigure were divided into six groups based on their ranking order for values of each variable. The error bar in each group denotes the mean value (black circle) and standard deviation (black bar) of normalization parameter.

The main finding in the previous section is that normalization parameters (m_1_, m_2_, and m_1_–m_2_) for high gamma are highly related to the discrepancy of spatial frequency between stimulus and sites’ preferences (Figs. 10e and 11b). This finding might be due to the fact that recording sites in A17 and A18 have different preferred spatial frequencies (preferred SF is lower for A18 than for A17^37^). We further checked whether this is the case. Based on the shift of the receptive field center of each recording site, we can determine whether a recorded site belongs to A17 or A18^38^. We measured the distance (in the unit of millimeter) of each site toA17/A18 border (A17: negative value; A18: positive value). Then the fitting parameters in the normalization model were compared with this distance index. For low gamma, there was no significant correlation between fitting parameters related to normalization and distance index (c_50_: r = − 0.15, p = 0.08; m_1_: r = − 0.07, p = 0.39; m_2_: r = − 0.05, p = 0.56; Fig. 12a–c), except that parameter b, which was related to the orientation selectivity, was significantly correlated with distance index (r = 0.28, p = 0.001; Fig. 12d). As we predicted, for high gamma, fitting parameters (c_50_, m_1_, and m_2_) were all significantly correlated with distance index (c_50_: r = − 0.38, p < 0.001; m_1_: r = − 0.4, p < 0.001; m_2_: r = − 0.18, p = 0.036; Fig. 12a–c). For MUA, the distance index was also significantly correlated with m_1_ (r = − 0.55, p < 0.001), but weakly correlated with c_50_ (r = − 0.19, p = 0.029) and not significantly correlated with m_2_ (r = 0.17, p = 0.05). Consistent with previous results which showed that Delta SF had different effect on m_1_–m_2_ (Fig. 10), the distance index was significantly correlated with m_1_–m_2_ for MUA (r = − 0.52, p < 0.001), but there was no significant correlation between distance index and m_1_–m_2_ for low gamma (r = − 0.02, p = 0.8) and high gamma (r = 0.06, p = 0.51). What’s more, the fitting parameters (c_50_, m_1_, m_2_, and m_1_–m_2_) under A17 were significantly different from those under A18 for high gamma (c_50_: p = 0.018; m_1_: p = 0.014; m_2_: p = 0.002; m_1_–m_2_: p = 0.03; Fig. 12). Interestingly, the distribution of m_1_–m_2_ for high gamma (Fig. 12e) showed a clear difference (similar to Fig. 10e) for A17 and A18: Most sites (19/36) showed m_1_–m_2_ < 0 is in A17, and the majority sites (72/101) located in A18 have m_1_–m_2_ > 0 (Also see consistent results in figure S9 for data where a single signal was good, n = 325 for LG; n = 375 for HG; n = 183 for MUA).

Taken together, the model parameters of low gamma, high gamma, and MUA have very different distribution patterns in brain areas 17 and 18. This result again implies that distinct neural mechanisms are behind low gamma, high gamma, and MUA, and normalization is region-specific, pathway-specific, and stimulus-specific**.**

## Discussion

In this work, we have systematically studied the response patterns of MUA and gamma oscillations induced by plaid stimuli with a descriptive model to unify normalization effects in both spiking activity and gamma oscillations. We then quantitatively compared normalizations in different neural signals. We demonstrated that gamma oscillations in cat visual cortex are mostly regulated by cross-orientation suppression but in a manner different from the regulation of spiking activity (MUA). Furthermore, we found that the model parameters for gamma oscillations were more diverse than those for MUA. Model parameters that capture diversities of low/high gamma and MUA showed distinct relationships with discrepancy of spatial frequency between stimulus and preference of sites. As for spatial transition on brain mapping from cat area 17 to 18, model parameters of low/high gamma and MUA also showed three distinct patterns. Overall, our results imply that the neural mechanisms for normalizations of gamma oscillations and MUA differ from each other.

### Comparisons between existing studies and the current work

Our study demonstrates that cross-orientation suppression of gamma oscillation is a common phenomenon in anesthetized cats. This finding of cross-orientation suppression of gamma oscillation is consistent with previous studies implemented in awake monkeys^21,22^ and humans^24^. However, no explanation or descriptive model has been used to account for this phenomenon on gamma oscillation.

Compared with previous studies, we have made progress in several directions. (1) We derived a spectrum-fitting procedure to capture the signature of the narrow-band gamma oscillation, which is quite different from studies on the broad-band component of the LFP^39–41^. Only a few studies have considered the separation of the narrow-band and broad-band oscillations^35,41^, which are quite important and necessary. (2) An interaction index was derived to quantify interaction effects between the responses induced by the two-component gratings used to generate the plaids^22^. Through this method, we were able to compare the response patterns among gamma oscillations and MUA. (3) We proposed a modified normalization model adapted to our data by adding and adjusting fitting parameters based on previous models^28,29^.

Our study revealed several interesting findings: first, the response patterns of the gamma oscillations for some sites showed cross-orientation facilitation instead of suppression. Second, although the response patterns of both gamma oscillations and MUA were different, they could be depicted by a unified normalization model. Third, the normalization parameters for gamma oscillations had a broader range than that for MUA.

### Comparison of the modified normalization model with other models

Normalization models^28,29^ have previously been provided to explain the cross-orientation suppression of spiking activity. We are curious about whether the existing normalization models^27,31^ could also explain the response patterns of gamma oscillations to plaids. The existing normalization model^29^ is expressed as:

$${R=R}_{max}\cdot \frac{{{c}_{1}}^{n}}{{{c}_{50}}^{n}+{{c}_{1}}^{n}{{+(k\cdot c}_{2})}^{n}}.$$

We directly applied this model to our data but found that it was not suitable for explaining our data (Figure S2a in supplementary information). This result implies that the gamma oscillations induced by the superimposed gratings were more complicated than assumed by the normalization model based on contrast gain control^42^.

Based on the assumption that the cross-orientation suppression of gamma oscillation may be due to the competition between the two-component gratings of the plaid^22^, we modified the previous normalization model (Modified model denotes as M_1_) by introducing a weighting variable (b) to combine the responses driven by the two-component gratings. Furthermore, according to the observation of our response patterns, we utilized two independent exponent variables (m_1_ and m_2_), rather than a common one^28^ (n), for the numerator and denominator in the normalization model. According to this way, another normalization model used in previous studies^27,31^ was also modified (M_2_). We applied the above two modified normalization models (M_1_, M_2_) to our response patterns of gamma oscillations and MUA, and compared the performance of M_1_ and M_2_ with our model (M_0_). The equations for these three models are:

$$\text{M}0{:}\; {{R}_{1}=R}_{max}\cdot \frac{{c}_{1}^{{m}_{1}}}{{{c}_{50}}^{{m}_{2}}+{({c}_{1}+{c}_{2})}^{{m}_{2}}}, \quad {{R}_{2}=R}_{max}\cdot \frac{{c}_{2}^{{m}_{1}}}{{{c}_{50}}^{{m}_{2}}+{({c}_{1}+{c}_{2})}^{{m}_{2}}}, \quad \text{R}=\text{b}\cdot {\text{R}}_{1}+(1-b)\cdot {R}_{2}.$$

$$\text{M}1{:}\; {{R}_{1}=R}_{max}\cdot \frac{{{c}_{1}}^{n}}{{{c}_{50}}^{n}+{{c}_{1}}^{n}{{+(k\cdot c}_{2})}^{n}}, \quad {{R}_{2}=R}_{max}\cdot \frac{{{c}_{2}}^{n}}{{{c}_{50}}^{n}+{{(k\cdot c}_{1})}^{n}{{+c}_{2}}^{n}}, \quad \text{R}=\text{b}\cdot {\text{R}}_{1}+(1-b)\cdot {R}_{2}.$$

$${{\text{M}2{:}\; R}_{1}=R}_{max}\cdot \frac{{c}_{1}^{{m}_{1}}}{{{c}_{50}}^{{m}_{2}}+{{c}_{1}}^{{m}_{2}}+{{c}_{2}}^{{m}_{2}}}, \quad {{R}_{2}=R}_{max}\cdot \frac{{c}_{2}^{{m}_{1}}}{{{c}_{50}}^{{m}_{2}}+{{c}_{1}}^{{m}_{2}}+{{c}_{2}}^{{m}_{2}}}, \quad \text{R}=\text{b}\cdot {\text{R}}_{1}+(1-b)\cdot {R}_{2}.$$

The goodness of fit of these three models was calculated for all the recording sites (Figure S2b–e), and Model M_2_ showed the lowest performance. We also compared the fit response with the real response for each site concerning the interaction index (details provided in the methods) for these three models (Figure S3). A bootstrap procedure was used to compare the correlation coefficients between the fit and real responses. The coefficient was higher with our model (M_0_) than with the other two models (Figure S4). In summary, our model (M_0_) showed the best performance of all three normalization models. Despite the good performance of our normalization model, it lacks a description of the neural circuit, and therefore cannot interpret the neural mechanism behind the response patterns of the gamma oscillation. In future work, more mechanistic models such as a model of dynamic systems based on the interactions between excitatory and inhibitory neurons should be created to obtain a full understanding of the changing effects of plaids on gamma oscillations.

### The characteristics of two distinct narrow-band gamma oscillations

Gamma oscillation is an outstanding feature of local field potentials (LFP) in the visual cortex, but the neural mechanisms and functions are still under debate. Early studies supported that gamma plays important roles in binding visual information^4,9^; however, growing evidence showed that gamma oscillation is stimulus dependent^2,43^ and not always visible^41,44,45^, which implies that gamma is a by-product of network activity and it may not be essential for cognitive functions. Consistent with early work^46,47^, we found two distinct gammas in the visual cortex. This is an interesting result for both hypotheses about the functional roles of gamma oscillations. For the hypothesis that supports gamma is a by-product of the neural network, how the neural network generates two narrow-band gamma oscillations is a challenging question. Moreover, for the hypothesis that support gamma is important for cognitive functions, the challenging question is to answer the different roles that the two gamma oscillations have. This paper is not intended to decide which sides are right, but the fact that gamma oscillations are regulated by normalization in a way different from MUA suggests that gamma oscillations are important signals for us to understand the neural network.

Unlike previous studies that reported the existence of two gamma oscillations in the spiking activity of cat visual cortex^47^, the gamma oscillations measured in our study were acquired by carefully separating the narrow-band components from the broad-band components of the LFPs. There are several reasons why we emphasize the importance of narrow-band gamma oscillations. The broad-band component of an LFP may imply spectral properties of the excitatory postsynaptic potentials or leakage of the high frequency components of the spiking activity into the LFP^48–50^. In contrast, the narrow-band oscillation in an LFP may reflect the interaction between the fast-spiking inter neurons and pyramidal cells^51^. The narrow-band gamma oscillation is quite different from the broad-band component of LFPs. Recent work^46^ demonstrates that two narrow-band gamma oscillations coexist in the LFP of primates, with two distinct frequency bands (slow gamma, 25–45 Hz, and fast gamma, 45–70 Hz). However, the frequency ranges of our low gamma (30–70 Hz) and high gamma oscillations (70–100 Hz) were higher than reported in the previous study. It has been demonstrated that the peak frequency of gamma oscillations depends on the anesthetization level^52,53^. Whether or not the difference between the frequency ranges was inherited from different mechanisms needs to be further investigated.

Our results showed that the characteristics of these two narrow-band gamma oscillations are distinct from each other. First, there was no significant correlation (r = 0.02, p = 0.78) between low gamma (LG) and high gamma (HG) in respect to the interaction index (Fig. 4c). This is an interesting result that plaid can induce two distinct response patterns for LG and HG simultaneously. Second, the fitting parameter (m_1_–m_2_) for low gamma is not significantly correlated with Delta SF, while (m_1_–m_2_) for high gamma is affected by specific spatial frequency. Finally, the diversity of (m_1_–m_2_) for high gamma (not low gamma) is region-specific for brain areas (A17/A18). These correlation analyses imply that low gamma and high gamma are regulated by different rules of normalization.

Interestingly, the model parameters (m_1_–m_2_) and m_2_ are widely distributed and strongly correlated with the Delta SF for high gamma. Our current study cannot fully reveal the neural mechanisms underlying the results of correlation and diversity for HG responses. Neural normalization might involve multiple neural circuitries, including feedforward signals, local recurrent connection, and feedback connections, similar to neural oscillations^43,45,54^. Characteristics of both normalization and oscillations are combined effects of these neural circuits^37^. We speculate that high gamma and its normalization is generated pre-cortically^29,47^. In the pre-cortical stage (retina or LGN), the X and Y cells generate their own high gamma with distinct spatial frequency preferences. Therefore, we see distinct values of (m_1_–m_2_) and m_2_ between A17 and A18, which leads to a strong correlation between Delta SF and (m_1_–m_2_). However, low gamma is generated in the cortical network, which combines the inputs of X and Y cells from LGN. The combination of multiple feed-forward inputs will weaken the relationship between normalization and spatial frequency preferences. A recent modeling work also implies that low and high gamma oscillations are different signatures for neural connections in different spatial scales^55^. In future work, in order to dissect different circuitry, it will be worth investigating normalizations and oscillations with plaids at different stimulus sizes and spatial frequency^27,28^. It is also worth building a dynamic model^56^ to further understand parameters in the descriptive normalization model behind these two gamma oscillations and their normalizations.

## Supplementary Information

Supplementary Information.

## Data Availability

The data and code used in our current study have not been deposited in a public repository because the electrophysiological data of cats were stored in self-customized. If one has specific request, contact the author for the data.
