# Supplementary material for: Superimposed gratings induce diverse response patterns of gamma oscillations in primary visual cortex
Source: Sci Rep. 2021 Mar 2;11:4941. doi: 10.1038/s41598-021-83923-5 (PMC7925546; doi:10.1038/s41598-021-83923-5)
Supplement: Supplementary file 1 — Supplementary Information. [file 41598_2021_83923_MOESM1_ESM.pdf]

Supplementary Information

Superimposed gratings induce diverse response patterns of gamma oscillations in primary visual cortex

Bin Wang, Chuanliang Han, Tian Wang, Weifeng Dai, Yang Li, Yi Yang, Guanzhong Yang, Lvyan Zhong, Yange Zhang, Yujie Wu, Gang Wang, Hongbo Yu, Dajun Xing

	Low Gamma (n=138)	High Gamma (n=138)	MUA (n=138)	Low Gamma (n=325)	High Gamma (n=375)	MUA (n=183)
Weak Grating (Median)	0.4	0.79	0.015	0.49	0.78	0.014
Weak Grating (CI)	[0.35 0.47]	[0.77 0.91]	[0.027 0.041]	[0.52 0.62]	[0.76 0.87]	[0.027 0.039]
Strong Grating (Median)	0.83	1.1	0.077	0.94	1.1	0.075
Strong Grating (CI)	[0.77 0.88]	[1.04 1.17]	[0.064 0.091]	[0.90 0.99]	[1.0 1.1]	[0.064 0.087]
Plaid (Median)	0.74	1.1	0.057	0.79	0.99	0.062
Plaid (CI)	[0.66 0.78]	[1.07 1.19]	[0.08 0.12]	[0.73 0.8]	[1.0 1.09]	[0.08 0.11]

Table S1. Summary of the distributions of responses (low gamma, high gamma, and MUA) induced by different stimuli (two gratings with 45% contrast and plaid with 90% contrast). The median and 95% confidence interval (CI) for all the responses induced by gratings and plaid were listed. Note that the values for gamma oscillations denote the relative gamma power which is same as the definition in figure 2. While, MUA was defined as the multi-unit activities under stimulus condition subtracted by those under blank condition.

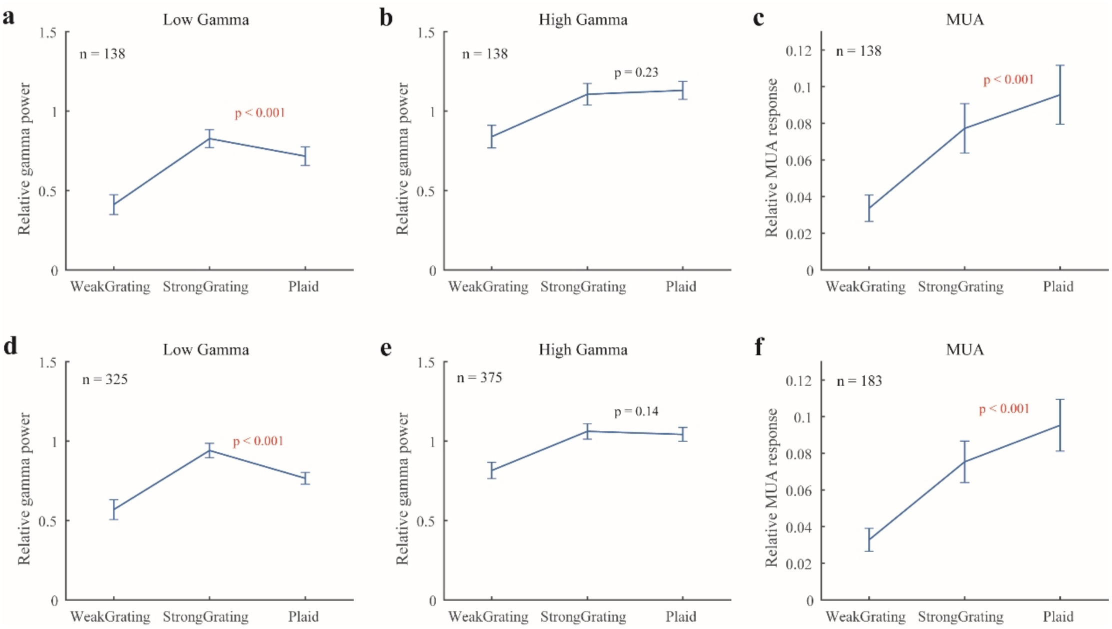

Figure S1. The summary responses for three signals under two kinds of dataset. Subfigure a shows the comparisons (mean and standard error of mean) among the low gamma power induced by three different stimuli for sites with all signals good (LG, n =138). Subfigures b and c are similar to a, but for high gamma and MUA respectively. Subfigures d-f are similar to a-c, but for sites with good low gamma (LG, n = 325), sites with good high gamma (HG, n = 375), and sites with good MUA (MUA, n = 183) respectively. Note that in each subfigure, strong grating denotes the grating with 45% contrast in the 49 plaids (same stimuli in Figure 3) that induced relatively strong response compared with the orthogonal grating (weak grating). The plaid denotes the superimposed gratings with 45% contrast. The p-value (T-test) accounts for the significant difference between strong grating and plaid responses. The relative gamma power is defined as the ratio between stimulus and blank power in logscale (same as the relative power in figure 2), while relative MUA response is defined as the difference between mean response of stimulus condition and that of blank condition.

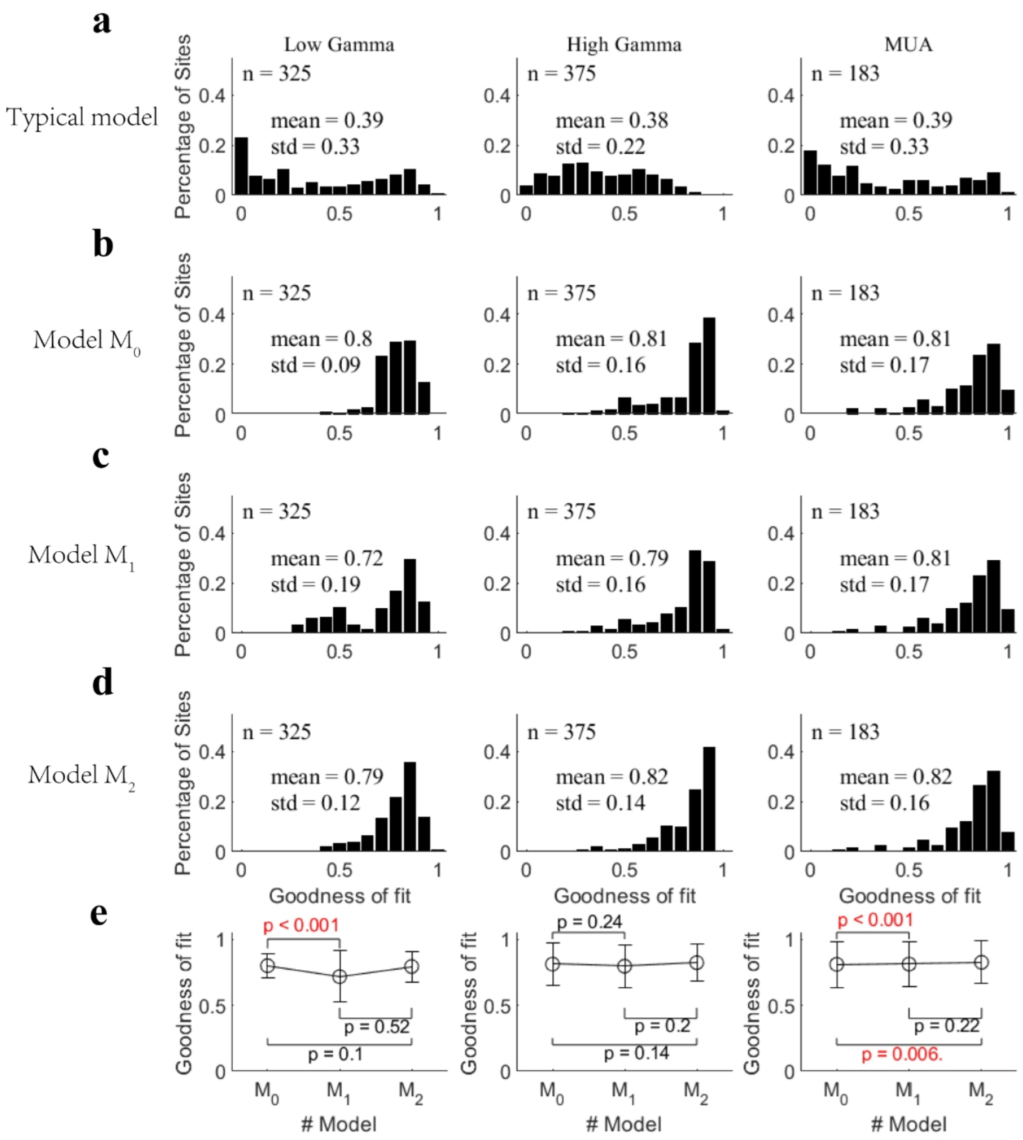

Figure S2. The comparison among four different normalization models[1]. Subfigure a shows the performance of typical model for explaining the response patterns under three signals (from left to right, low gamma, high gamma, and MUA). Subfigures b-d are similar to a, but for other three modified normalization models (M_0, M_1, M_2). Since the typical model is not suitable for our data, the comparisons among other three models were calculated in subfigure e. The error bar denotes the mean and standard deviation. Note that the equations of above models are listed below.

Typical model: $R = R_{max} \cdot \frac{c_1{}^n}{c_{50}{}^n + c_1{}^n + (k \cdot c_2)^n}$.

M_0: $R_1 = R_{max} \cdot \frac{c_1^{m_1}}{c_{50}{}^{m_2} + (c_1 + c_2)^{m_2}}, R_2 = R_{max} \cdot \frac{c_2^{m_1}}{c_{50}{}^{m_2} + (c_1 + c_2)^{m_2}}$, $R = b \cdot R_1 + (1 - b) \cdot R_2$.

M_1: $R_1 = R_{max} \cdot \frac{c_1^{m_1}}{c_{50}{}^{m_2} + c_1{}^{m_2} + c_2{}^{m_2}}, R_2 = R_{max} \cdot \frac{c_2^{m_1}}{c_{50}{}^{m_2} + c_1{}^{m_2} + c_2{}^{m_2}}$, $R = b \cdot R_1 + (1 - b) \cdot R_2$.

M_2: $R_1 = R_{max} \cdot \frac{c_1{}^n}{c_{50}{}^n + c_1{}^n + (k \cdot c_2)^n}, R_2 = R_{max} \cdot \frac{c_2{}^n}{c_{50}{}^n + (k \cdot c_1)^n + c_2{}^n}, R = b \cdot R_1 + (1 - b) \cdot R_2$.

Note that the statistical significances were calculated by bootstrap method. The significant results were highlighted

with red fonts.

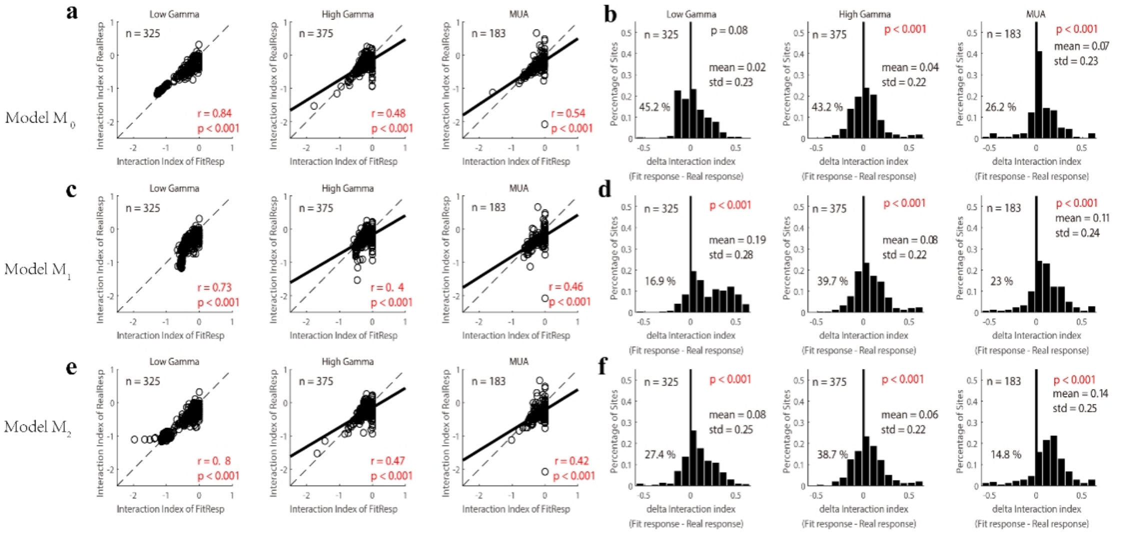

Figure S3. The comparison among interaction index of fit response and real response for two gamma oscillations and MUA. a) shows the scatter diagrams of interaction index of fit response and that of real response for low, high gamma and MUA through modified normalization model M_0. Pearson's correlation was used to quantify the linear correlation between these two indices. b) shows the histogram of the difference between interaction index of fit response and that of real response (delta interaction index). We used two tailed t-tests to test whether the distribution of delta interaction is significantly biased from zero. Similar to a and b, c and d repeat the performance of Model M_1 in respect of the linear correlation between interaction index of fit and real response. e and f are also similar to a and b, but for Model M_2. Note that the delta interaction indices in these three models are quite low, and there exist strong correlation between the interaction index of fit and real response. The statistically differences among degree of linear correlation of these three models need to be further tested. Linear regression was calculated for correlation measurement (black solid line).

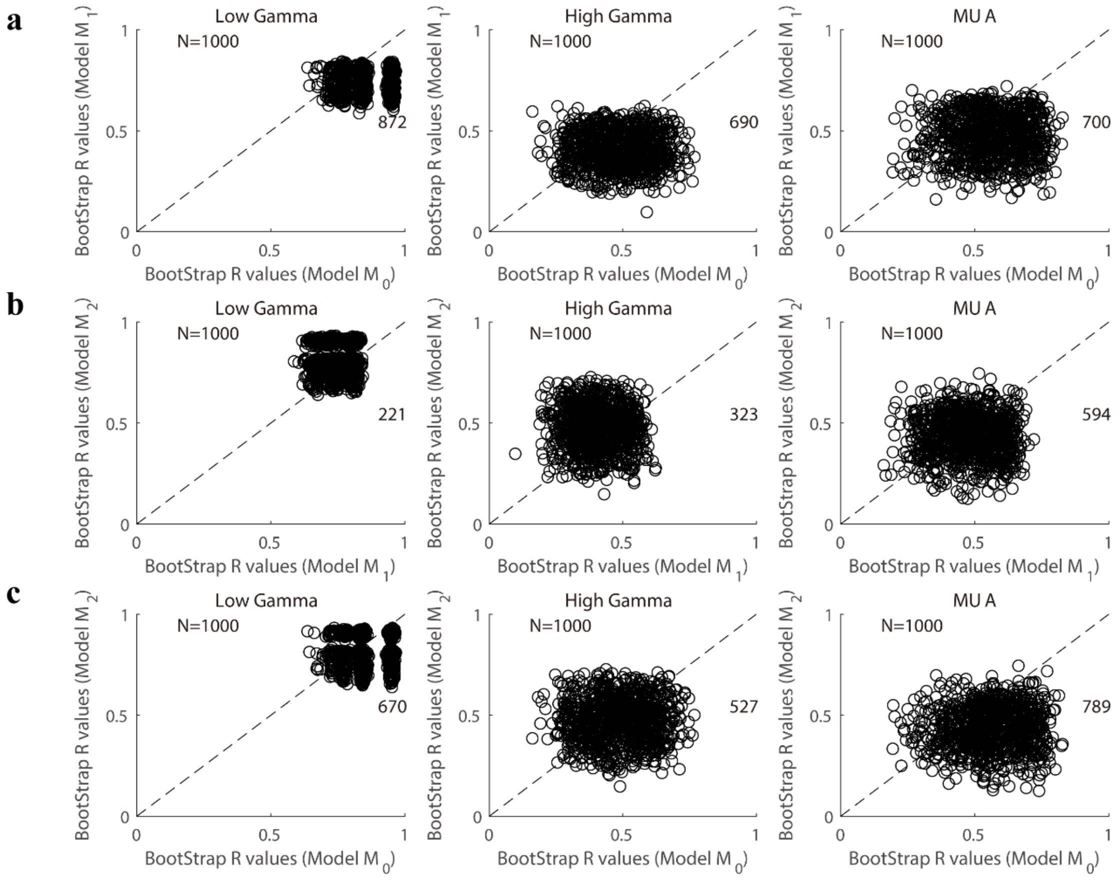

Figure S4. The statistically comparison among the correlation coefficient (between fit response and real response) of three modified models (M_0, M_1, M_2) through Bootstrap tests. We randomly select n data points from the raw data (n) with replacement and calculate the pairwise linear correlation coefficient between the real response and fit response with a given modified normalization model for three signals (low gamma, high gamma, and MUA). Then repeat this operation 1000 times with different modified normalization models (M_0, M_1, M_2). We can get the linear correlation coefficients (R) of 1000 samples for three models (M_0, M_1, M_2). a) shows the scatter diagrams of R of M_0 and that of M_1 for three signals (from left to right: low gamma, high gamma and MUA). Note that R of M_0 is higher than that of M_1 for most samples (n=872 for low gamma; n= 690 for high gamma; n=700 for MUA). b is similar with a, but for the comparison between M_1 and M_2. Similar to a, c shows the comparison between M_0 and M_2 in respect of R. Taken together, the correlation coefficient of modified model M_0 is higher than other two models for most samples.

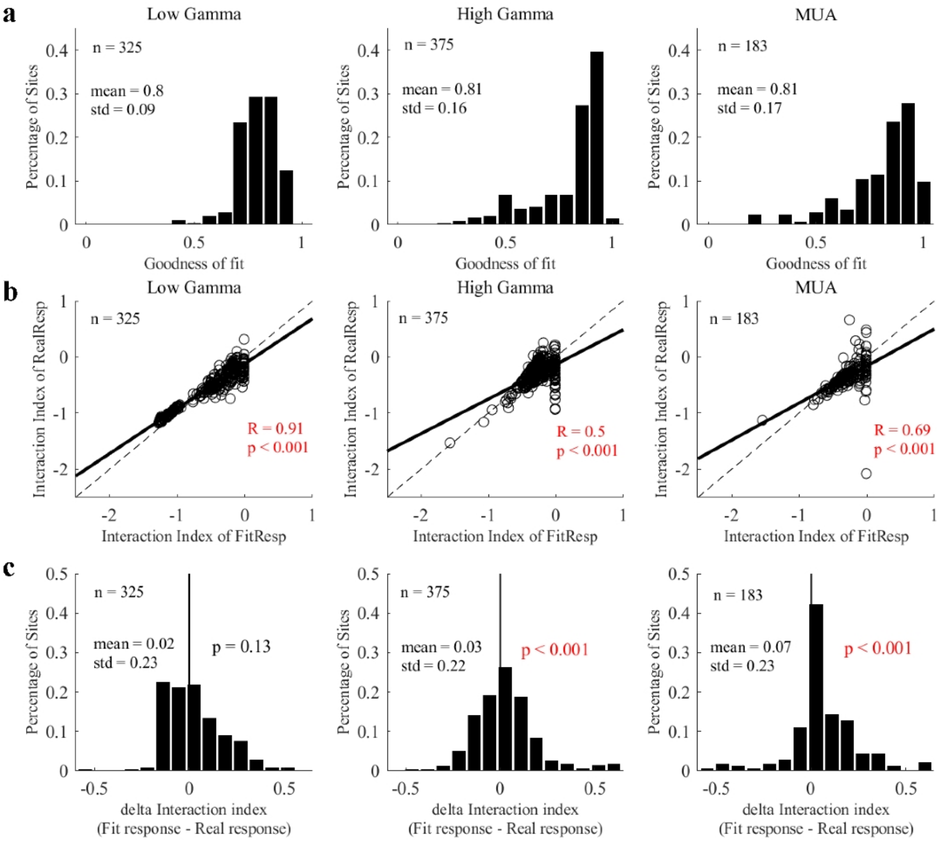

Figure S5. Strong correlations between the interaction index of fit responses and that of real responses. a) presents histograms of the goodness of fit of the normalization model for sites with individual signal good (low gamma, n=325; high gamma, n = 375; and MUA, n = 183). b) shows comparisons between the interaction index of real responses and that of the fit responses for sites with individual signal good. Linear regression was calculated to confirm the linear correlation (the black solid line). c) presents histograms of the differences between the fit responses and real responses. Note that Pearson's correlation was used to test the significance of the relationship between the two indices, and the bootstrap method was used to test whether the distribution of the delta interaction index was significantly different to 0. All the significant correlations were labeled as red fonts.

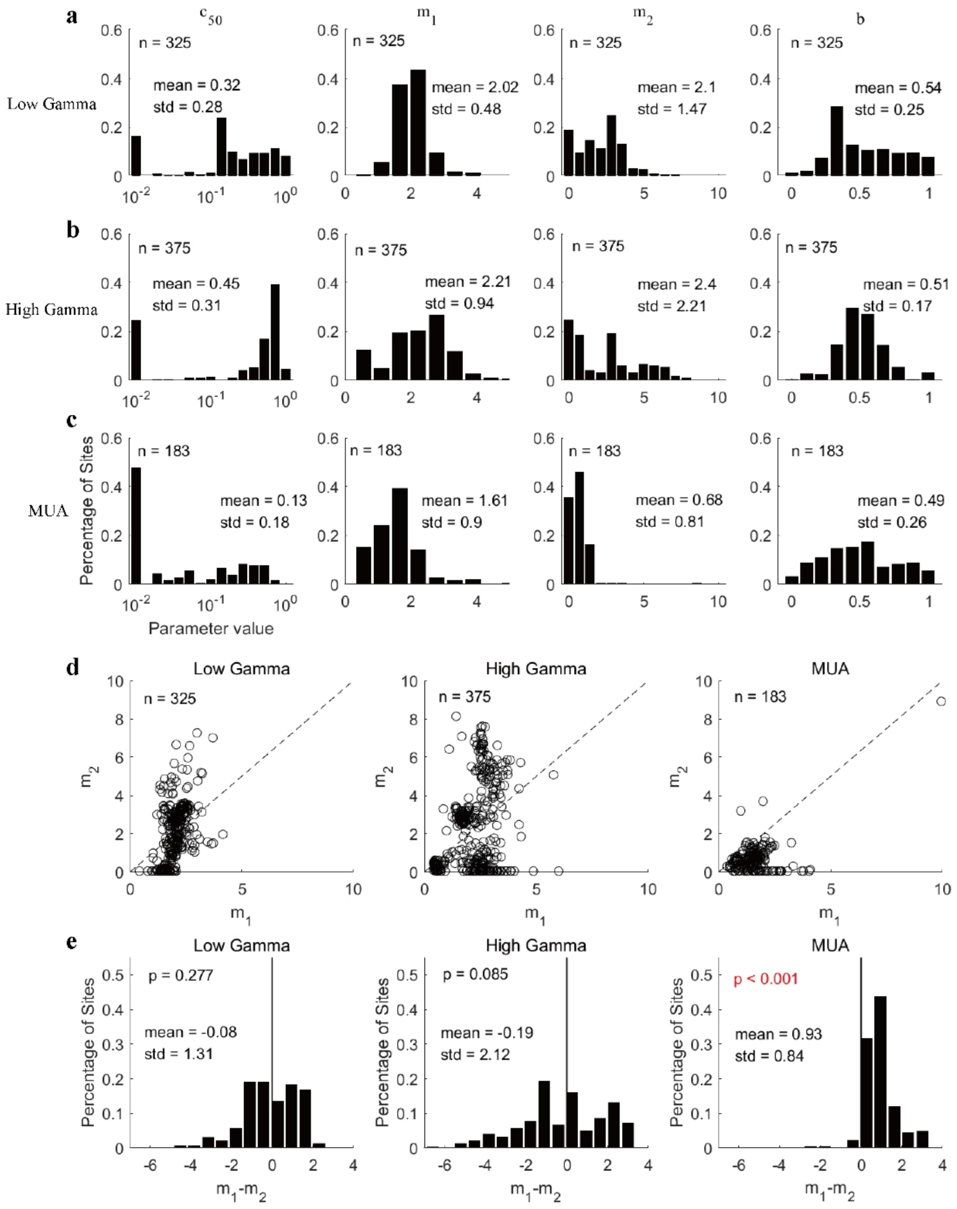

Figure S6. Analysis of the fitting parameters in the normalization model for three kinds of sites with three signals good respectively (low gamma, n =325; high gamma, n = 375; MUA, n = 183). a) shows histograms of various fitting parameters (c_{50}, m_1, m_2, b) for low gamma. Subfigures b and c are similar to a, but for high gamma and MUA respectively. d) presents scatter diagrams of the parameters m_1 and m_2 for three signals. e) shows histograms of the difference between m_1 and m_2 for these three signals. The bootstrap method was used to test whether the distributions were significantly biased from 0 (black solid line). All the significant correlations were labeled as red fonts.

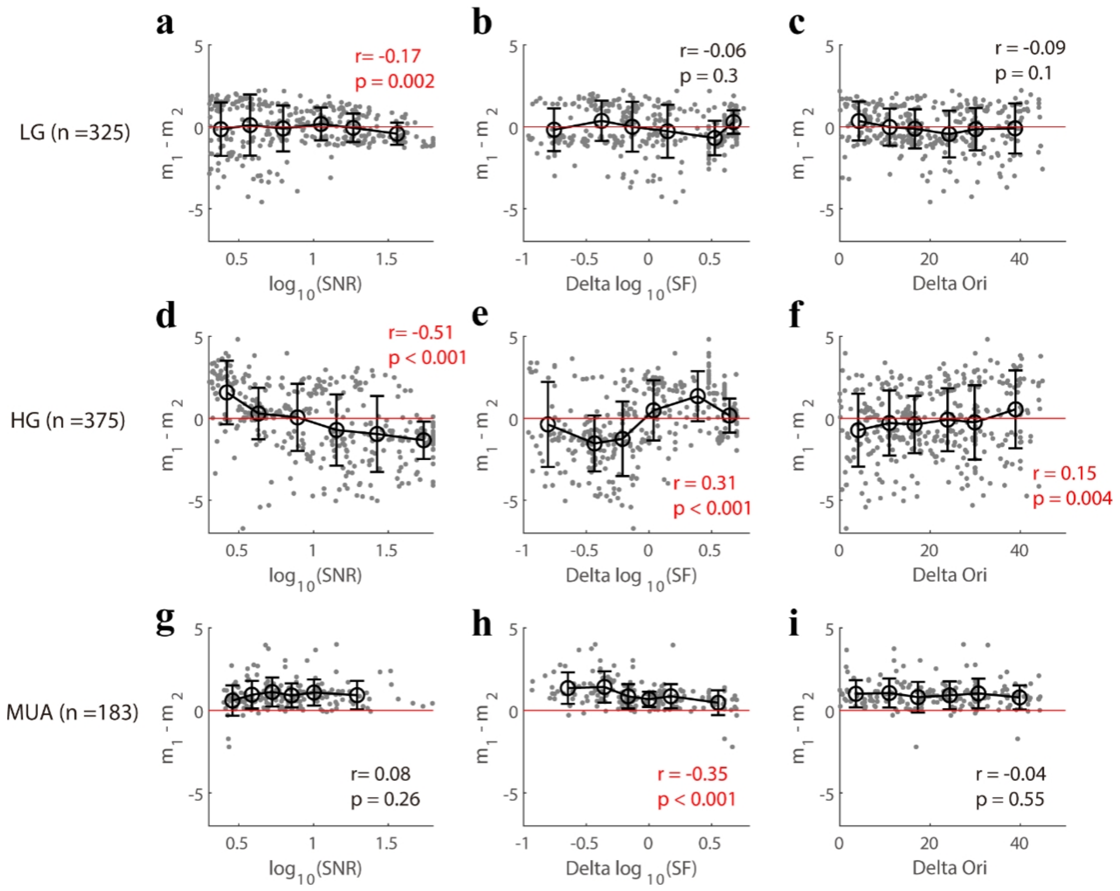

Figure S7. The relationship between several factors (SNR, Delta SF, and Delta Ori) and fitting parameter (m_1-m_2) for three signals (low gamma, high gamma, and MUA). For sites with good low gamma (LG, n = 325), a) shows the scatter plot of signal-noise ratio (SNR) and (m_1-m_2); b) presents scatter plot of the Delta SF (the ratio of the chosen stimulus spatial frequency and preferred spatial frequency of individual sites) and m_1-m_2; c) shows the scatter plot of Delta Ori (the difference between the chosen stimulus orientation and preferred orientation of individual sites) and (m_1-m_2). The data points in subfigures (a-c) were divided into six groups based on their ranking order for values of each variable. The error bar in each group denotes the mean value (black circle) and standard deviation (black bar) of (m_1-m_2). Subfigures (d-i) are similar to (a-c), but (d-f) are for sites with good high gamma (HG, n = 375), while, (g-i) are for sites with good MUA (n = 183). Note that Spearman's correlation was used to check their relationship. All the significant correlations were labeled as red fonts.

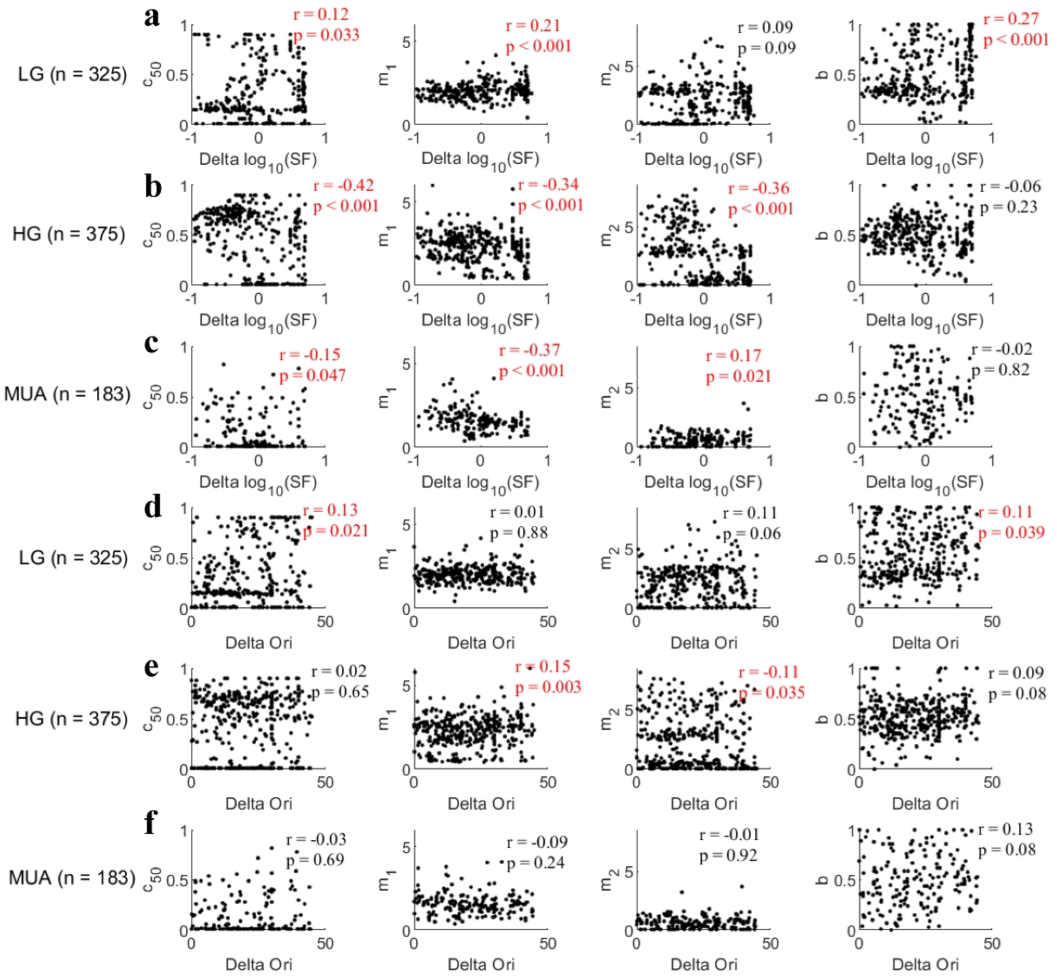

Figure S8. The relationship between fitting parameters and stimulus factors (Delta SF and Delta Ori). Subfigure a presents the relationship between Delta SF and the four fitting parameters (from left to right: c_{50}, m_1, m_2, b) for low gamma (LG, n = 325 sites). Subfigure b and c are similar to a, but for high gamma (HG, n = 375 sites) and MUA (n = 183) respectively. Subfigure d shows the scatter plot of Delta Ori and the four fitting parameters (from left to right: c_{50}, m_1, m_2, b). Note that Spearman's correlation was used to account for the relationship between the stimulus factors and the fitting parameters. Subfigure e and f are similar to d, but for high gamma and MUA respectively. All the significant correlations were labeled as red fonts.

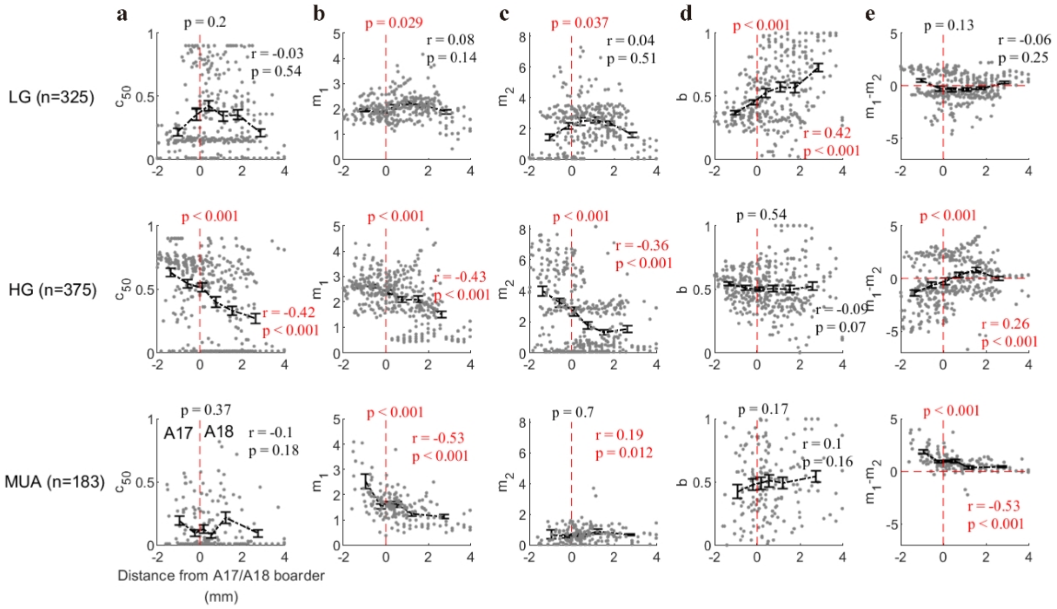

Figure S9. The correlation between brain areas (A17 vs A18) and fitting parameters. The distance from the border between Area 17 and Area 18 was used to represent the spatial position of the individual recording site. The A17/A18 border was determined through the previous work[2]. Subfigure a shows the relationship between the distance from A17/A18 border (distance index) and fitting parameter c_{50} for three signals (from top to bottom: low gamma, high gamma, and MUA). Subfigures (b-d) are similar to a, but they account for the relationship between distance index and fitting parameters m_1, m_2, b, and m_1-m_2 respectively. Spearman's correlation was used to account for correlation measurement. The p-values (T-test) located above red dashed linesin each subplot represent the statistical significances for whether parameters in brain Area 17 are significantly different from Area 18. Note that the sites with negative distance index are located in A17, while the positive distance index means A18. The data points in each subfigure were divided into six groups based on their ranking order for values of each variable. The error bar in each group denotes the mean value (black circle) and standard deviation (black bar) of normalization parameter.

Supplementary Reference

1 Heuer, H. W. & Britten, K. H. Contrast dependence of response normalization in area MT of the rhesus macaque. *Journal of neurophysiology* 88, 3398-3408 (2002).

2 Zheng, L. & Yao, H. Stimulus-entrained oscillatory activity propagates as waves from area 18 to 17 in cat visual cortex. *PloS one* 7, e41960 (2012).
